# Exploring the mechanism of olfactory recognition in the initial stage by modeling the emission spectrum of electron transfer

**DOI:** 10.1371/journal.pone.0217665

**Published:** 2020-01-10

**Authors:** Shu Liu, Rao Fu, Guangwu Li

**Affiliations:** 1 Department of Anatomy, Anhui Medical University, Hefei, Anhui, China; 2 Department of Anatomy, School of Medicine, Sun Yat-sen University, Guangzhou, Guangdong, China; University of Calgary, CANADA

## Abstract

Olfactory sense remains elusive regarding the primary reception mechanism. Some studies suggest that olfaction is a spectral sense, the olfactory event is triggered by electron transfer (ET) across the odorants at the active sites of odorant receptors (ORs). Herein we present a Donor-Bridge-Acceptor model, proposing that the ET process can be viewed as an electron hopping from the donor molecule to the odorant molecule (Bridge), then hopping off to the acceptor molecule, making the electronic state of the odorant molecule change along with vibrations (vibronic transition). The odorant specific parameter, Huang–Rhys factor can be derived from *ab initio* calculations, which make the simulation of ET spectra achievable. In this study, we revealed that the emission spectra (after Gaussian convolution) can be acted as odor characteristic spectra. Using the emission spectrum of ET, we were able to reasonably interpret the similar bitter-almond odors among hydrogen cyanide, benzaldehyde and nitrobenzene. In terms of isotope effects, we succeeded in explaining why subjects can easily distinguish cyclopentadecanone from its fully deuterated analogue cyclopentadecanone-d28 but not distinguishing acetophenone from acetophenone-d8.

## Introduction

The sense of smell (olfaction) is vital to the survival. A great progress has been made in understanding the physiological and biochemical basis of olfaction, but it remains elusive regarding the primary reception mechanism in the initial stage. One argument is associated with the “lock and key” theory [1–4], it has been widely believed that odorant molecules bind to specific receptors through conventional molecular interactions, leading to a conformational change in the receptor that activates intracellular signals. However, this theory could not predict the odor character of molecules and rational odorant design due to the large number of ORs and the breadth of OR tuning [5]. An alternative argument is the “vibration” theory [6–10], which was recently brought to prominence by Luca Turin [11], suggested that olfaction is a spectral sense and that an olfactory event is triggered by electron transfer (ET) within an olfactory receptor (OR). Since vibrational theory was put forward, the debate has continued [12–28]. Horsfield et al.[29] made an attempt to bring together the various theories into a single formalism regarding the olfactory recognition step. Hoehn et al.[30] reviewed the status of the vibrational theory of olfaction, both the historical iterations and the present iteration, to present the vital findings and models used during the validation and examination of the contemporary vibrational theory of olfaction.

Although the vibrational theory was successfully to be used to predict the smells of certain well-documented odorants [13], it has not been widely accepted, the opponents of vibration theory think that vibration theory has failed in multiple instances. For example, Keller and Vosshall [14] performed a series of psychophysical experiments to test several claims made by Turin [11], and they found no evidence to support the vibrational theory. A convenient way to test the vibrational theory is validation of the odor character differences between deuterated and nondeuterated odorant isotopomers. One of the experiments by Vosshall indicated that human panelists cannot discriminate the isotopologues of acetophenone [14].

We think that these negative pieces of evidence are insufficient to prove the implausibility of vibrational theory, especially they cannot prove that there's no ET in the OR. The previous model of vibrational theory may not explain the phenomenon of isotope effect well, because according to Turin’s theory [11], replacement of hydrogen with deuterium reduces the C-H stretch frequency from the 3000 cm^-1^ region to the 2200 cm^-1^ region, making the odor character difference between acetophenone isotopomers easily detectable [17].

**Fig 1:**
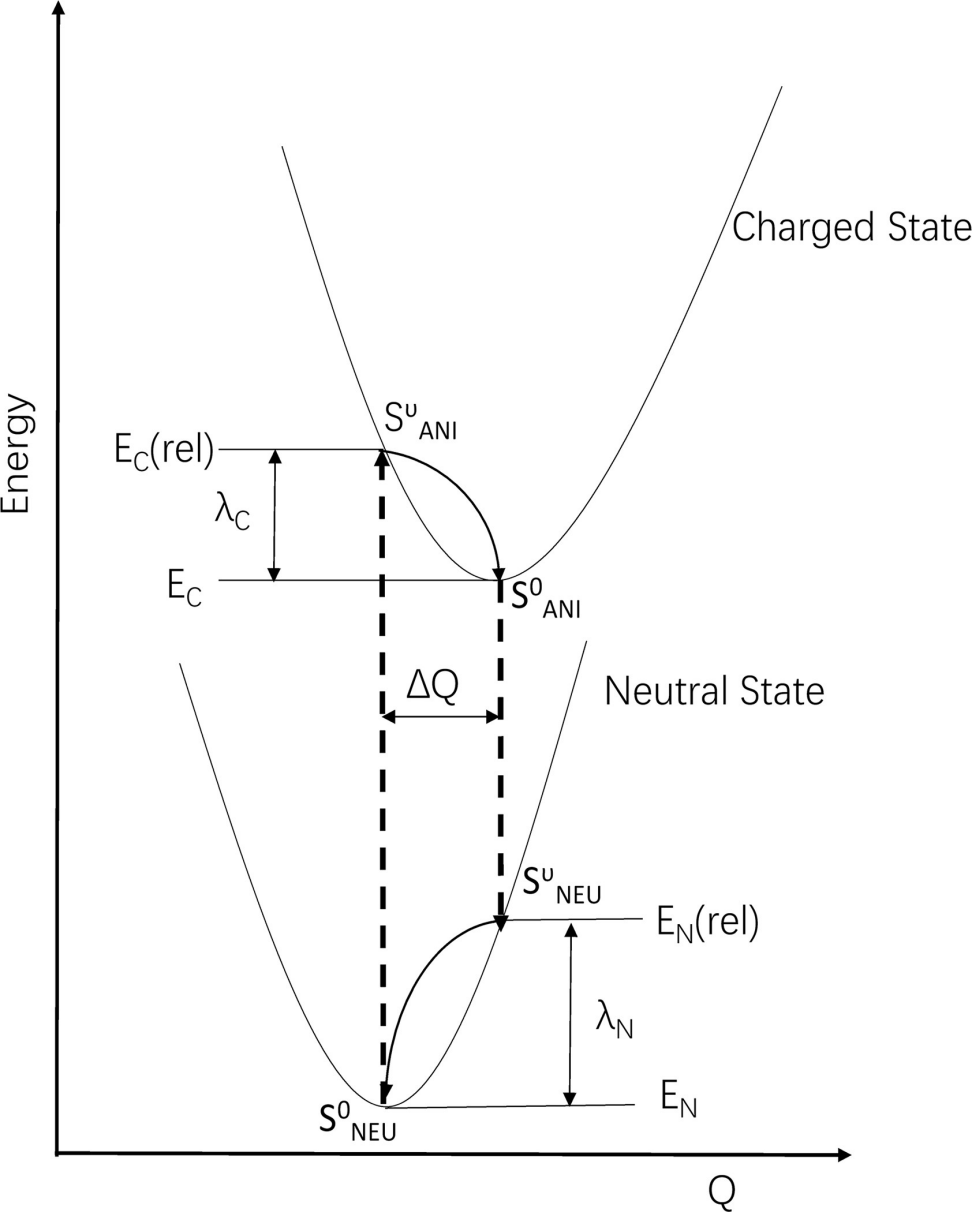
Sketch of the potential energy surfaces for the neutral state and charged state, showing the four vibronic states, $S_{\mathrm{NEU}}^{0},S_{ANI}^{\upsilon },S_{\mathrm{ANI}}^{0}$ and $S_{\mathrm{NEU}}^{\upsilon }$, the vertical transitions (dashed lines), the normal mode displacement ΔQ, and the relaxation energies λ_N_ and λ_C_.

In the present work, we study the feasibility of ET via the odorant molecule within the OR. Once a suitable odorant molecule enters the binding pocket of the receptor and docks successfully, i.e., the shape fit and the orientation are correct according to van der Waals and electrostatic interactions, the odorant molecule may act as a bridge (B) molecule between the D and A molecules and forms the so-called Donor–Bridge–Acceptor (DBA) system. If the lowest unoccupied molecular orbital (LUMO) of the B molecule is approximately resonant with the LUMOs of the D and A molecules, an extra electron may jump from the D molecule to the B molecule, where it stays for a while before hopping off to the A molecule [31]. The odorant molecule (B) simultaneously changes its electronic state with an excitation of one or more phonons; we refer to this combination of vibrational and electronic transitions as the vibronic transitions. As illustrated in Fig 1, during the electron transfer process, the odorant molecule undergoes four vibronic states, $S_{\mathrm{NEU}}^{0}\to S_{ANI}^{\upsilon }\to S_{\mathrm{ANI}}^{0}\to S_{NEU}^{\upsilon }$, representing a neutral state with a ground vibrational state, anionic state with vibrational excitation, anionic state with a ground vibrational state and neutral state with vibrational excitation, respectively. With electron absorption, the odorant molecule starts in the vibronic state $S_{\mathrm{NEU}}^{0}$ and finish in $S_{\mathrm{ANI}}^{\upsilon }$; with electron emission, i.e., an extra electron jump from the odorant molecule (B) to A molecule, the B molecule starts in $S_{\mathrm{ANI}}^{0}$ and finish in $S_{\mathrm{NEU}}^{\upsilon }$.

Up to now, no OR has been crystallized yet, it is unknown where the electron donor (D) site and acceptor (A) site are within the OR, some parameters (the hopping integral between donor and acceptor states, the free energy and the intermolecular reorganization energy) of ET rates possess a higher degree of uncertainty, it is difficult to investigate what conditions lead to high ET rates, and what do not. For the weak interactions (van der Waals and electrostatic interactions) between the B molecule and the D and A molecules, we consider that the electronic coupling of the odorant molecule to the D (A) molecule is unlikely to modify the free molecule properties. Therefore, we only need to consider the individual odorant molecule to study the vibronic transition properties of the odorant, without involving the odorant molecule B and the D (A) molecule as a complex entity, so that the specific parameters (the vibrational frequency ω and Huang-Rhys factor $\bar{S}$) of ET rates can be derived from ab initio calculations.

Huang–Rhys factor $\bar{S}$ provides a quantitative description of electron-vibrational coupling property, which can be used to model the absorption and emission ET spectrum. The intensity of the emission spectrum is directly related to the Franck–Condon (FC) factors between the vibrational energy levels of the corresponding charging state [32], maximally contribute to the electron transfer from the odorant molecule to the acceptor molecule, which is proposed to be responsible for protein activation. If an electron transfer across the odorant within the OR and the vibronic transition occurs, the ET spectra of the odorants (after convolution) should correlate with their odor character. The band number, width and position of ET spectrum may be responsible for molecule’s odor character. Via extensive comparative spectral analysis, we revealed that the emission spectrum of ET could act as an objective evaluation criterion for odor character.

Two different tests were used to validate correlation between the emission spectrum of ET and the odor character of molecules as following described: (i) To explore why some odorants have different structures but possess similar smells, such as hydrogen cyanide (HCN), benzaldehyde and nitrobenzene. To be noted, the reason why HCN possesses bitter almond odor character has not yet been reasonably interpreted by “lock and key” theory. We simulated the ET spectra of these odorants, finding that the emission spectra were highly overlapped at a certain vibrational frequency. (ii) Isotope effects on odor character have been controversial between supporters and opponents of vibrational theory. The experimental studies of Gane et al.[21] showed that subjects are incapable of distinguishing acetophenone and its fully deuterated analogue acetophenone-d8 but can easily distinguish between cyclopentadecanone and cyclopentadecanone-d28. In this study, we were able to perfectly interpret this previous experimental results by comparing the ET emission spectrum of one molecule with its deuterated analogue.

## Methods

All quantum chemical calculations were performed using Gaussian 09 [33] and analyzed using Multiwfn [34]. For seven odorant molecules of interest: HCN, benzaldehyde (C_6_H_5_CHO), nitrobenzene (C_6_H_5_NO_2_), acetophenone (C_8_H_8_O), acetophenone-d8 (C_8_D_8_O), cyclopentadecanone (C_15_H_28_O), and cyclopentadecanone-d28 (C_15_D_28_O), we first perform geometry optimizations of the neutral states using the B3LYP density functional [35–37] with the 6-31G basis set [38,39] in a vacuum; the same functional and basis sets were then used to perform geometry optimizations of the anionic states at the neutral equilibrium geometry. Subsequently, single-point calculations were performed using a more flexible basis set augmented with diffuse functions (6–311+G**) [40,41]. IR spectra were generated by application of a broadening factor of 4 cm^-1^ and compiled for presentation using Excel. The intramolecular reorganization energies and the Huang-Rhys factors $\bar{S}$ were computed using a modified version of the DUSHIN program developed by Reimers [42].

### Calculation of the intramolecular reorganization energy and Huang–Rhys factor

The reorganization energy λ represents the changes to the nuclear positions corresponding to the charge movement; the total reorganization energy λ includes the intramolecular reorganization energy (λ_i_) and the external reorganization energy (λ_S_) components.

The intramolecular reorganization energy (λ_i_) consists of two terms related to the geometry relaxation energies upon going from the neutral molecular state to the charged (cationic or anionic) state and vice versa. As sketched in Fig 1, the overall intramolecular reorganization energies are the sum of the reorganization energies:

$$\lambda_{i}=E_{N}+E_{C}$$ (1)

The terms λ_N_ and λ_C_ were computed directly from the adiabatic potential-energy surfaces as [43–48]:

$$\lambda_{N}=E_{N}(rel)-E_{N}$$ (2)

$$\lambda_{C}=E_{C}(rel)-E_{C}$$ (3)

Here, E_N_(rel) and E_C_(rel) are the energies of the neutral state in the optimized (relaxed) geometry of a charged molecule and the energies of a charged state in the optimized geometry of a neutral molecule, respectively; E_N_ and E_C_ are the energies of the neutral state in the optimized geometry of a neutral molecule and the energies of the charged state in the optimized geometry of a charged molecule, respectively.

Within the harmonic-oscillator approximation, λ_N_ and λ_C_ may be partitioned into contributions from each vibrational mode according to:

λN=∑j=1Nυλj(N),λj(N)=ℏωj(N)S¯j(N),S¯j(N)=ωj(N)ΔQj(N)22ℏ (4)

λC=∑j=1Nυλj(C),λj(C)=ℏωj(C)S¯j(C),S¯j(C)=ωj(C)ΔQj(C)22ℏ (5)

where n_v_ is 3n-6 (if nonlinear) or 3n-5 (if linear) independent vibrational motions when a molecule has n atoms; *ћ* is the reduced Planck constant; ω_j_ represents the vibrational frequency; and ΔQ_j_ represents the displacement along normal mode j between the equilibrium positions of the two electronic states of interest. We have included in Eqs (4) and (5) a representation of the reorganization energy in terms of the Huang–Rhys factors ${\bar{S}}_{j}$, and we note that the Huang–Rhys factors are directly related to the ΔQ_j_ terms.

The first issue in calculating the Huang–Rhys factors in Eqs (4) and (5) arises from the fact that each vibrational wave function is expressed in a different set of normal coordinates. The numerical procedure consists of the following steps: First, the standard rectangular normal modes Q_N_ and Q_C_ are obtained as a linear combination of Cartesian displacements:

$$Q_{N}=L_{N}^{T}M^{1/2}\left(x_{N}-x_{N}^{\left(0\right)}\right)$$ (6)

$$Q_{C}=L_{C}^{T}M^{1/2}\left(x_{C}-x_{C}^{\left(0\right)}\right)$$ (7)

where M is the (3n×3n) diagonal matrix whose nonzero elements are the masses of the atoms associated with each Cartesian coordinate. The vectors $x_{N}^{\left(0\right)}$ and $x_{C}^{\left(0\right)}$ correspond to the equilibrium Cartesian coordinates on the adiabatic potential surfaces of the neutral and charged states, respectively. L_N_ and L_C_ are (3n×n_v_) transformation matrices from Cartesian coordinates to normal coordinates, containing the set of 3n Cartesian coordinates x_N_ and x_C_, and the n_v_ vibrational eigenvectors of the appropriate mass-weighted Hessian (second derivative of the molecular potential energy) matrices, respectively.

Then, using standard rectilinear normal modes, the normal mode displacements ΔQ_N_ are obtained by projecting the displacements Δx_N_ = $x_{C}^{\left(0\right)}$—$x_{N}^{\left(0\right)}$ from the neutral state to the charged state onto the normal modes of the neutral state vectors:

$${\Delta Q}_{N}=L_{N}^{T}M^{1/2}\left(x_{C}^{\left(0\right)}-x_{N}^{\left(0\right)}\right)$$ (8)

The normal mode displacements ΔQ_C_ are obtained by projecting the corresponding displacements onto the normal modes of the charged state vectors:

$${\Delta Q}_{C}=L_{C}^{T}M^{1/2}\left(x_{N}^{\left(0\right)}-x_{C}^{\left(0\right)}\right)$$ (9)

### Calculation of the Duschinsky rotation matrix

The normal modes of one electronic state are no longer orthogonal to the normal modes of the other electronic state, generally called the Duschinsky rotation effect [49,50]. The two sets of normal modes are approximately related by the linear transformation in Eq (10) [49]:

$$Q^{\prime }=JQ+\Delta Q$$ (10)

Here, Q’ and Q represent the normal coordinate vector of the initial and the final electronic state, respectively. In Eq (10) and hereafter, we use a prime to label the initial state quantities. ΔQ is the column vector of the displacement of the potential energy surfaces (PESs) of the two electronic states along the normal coordinates mentioned before, and J is called the Duschinsky rotation matrix and represents the mixing of the normal modes of the two states during the transition. This linear transformation is generally a good approximation when the molecule does not undergo a noticeable distortion during the transition. For a simple model with two vibrational modes, Eq (10) can be written as:

(Q1′Q2′)=(cosθ−sinθsinθcosθ)(Q1Q2)+(ΔQ1ΔQ2) (11)

Here, θ is the rotational angle. ΔQ_1_ and ΔQ_2_ are the displacements for mode 1 and mode 2, respectively. If the optimized geometries and normal coordinates of both states are available, ΔQ can be calculated with Eqs (8) and (9). J be calculated as:

$$J={\left(L^{\prime }\right)}^{T}L$$ (12)

In the present work, we use the DUSHIN program to calculate the Duschinsky rotation matrix J for those odorant molecules. A job is launched by the following command:

./dushin dushin.dat

The main control input is read from the file dushin.dat in each directory. The structure of odorant molecules in the neutral state was optimized at the B3LYP/6-31G level, and the charge and spin multiplicity were set to 0 and 1, respectively. The same functional and basis sets were then used to perform geometry optimizations of the anionic state at the neutral equilibrium geometry. Here, the charge and spin multiplicity were set to -1 and 2, respectively. Then, we calculate the vibrational frequencies with the obtained structure and obtain the Gaussian 09 output files and .fchk files.

Then, we appropriately write a dushin.dat file. For the first item in the first line in the dushin.dat file, we set its value to 1, i.e., we use the orthogonal curvilinear coordinates for analysis. After that, the dushin.dat file, two Gaussian output files and two .fchk files are placed in the current directory, and the DUSHIN program is run.

The main summary output is sent to dushin.out, containing the frequencies, normal modes, Duschinsky matrices, intramolecular reorganization energies and mode-projected displacements (for use in generating the Huang–Rhys factors).

### Modeling the electron transfer spectrum

In molecular spectroscopy, the Franck–Condon factors (FCFs), related to the Huang–Rhys factors $\bar{S}$, determine the probability of specific electronic-vibrational transitions that give rise to vibrational replicas in the absorption and emission spectra [51]. The FC factor is the square of the Franck–Condon integral (FCI), which is the vibrational overlap integral between two vibrational levels in different electronic states:

$${FCF}_{\left(\mu ,\upsilon \right)}={FCI}_{\left(\mu ,\upsilon \right)}^{2}={\left|\left\langle \chi_{\mu }(Q^{\prime })|\chi_{\upsilon }(Q)\right\rangle \right|}^{2}$$ (13)

Here, Q’ and Q are the normal coordinates of the initial and final electronic state, μ and υ represent the initial and final vibrational quantum numbers (phonons), and Χ_μ_(Q’) and Χ_υ_(Q) are the corresponding vibrational wave functions.

In the framework of the Born–Oppenheimer and Franck–Condon approximations, the shape of a band is governed by the FCI, and several methods have been proposed to calculate multidimensional FC integrals and FC factors [52–54]:

$${FCF}_{\left(\mu ,\upsilon \right)}={FCI}_{\left(\mu ,\upsilon \right)}^{2}=exp\{-\overset{-}{S}\}{\bar{S}}^{\left(\upsilon -\mu \right)}\frac{\mu !}{\upsilon !}{\left[L_{\mu }^{\left(\upsilon -\mu \right)}(\bar{S})\right]}^{2}$$ (14)

where $L_{\mu }^{\left(\mu ‐\upsilon \right)}$ is a Laguerre polynomial. The calculations are considerably simplified when Duschinsky mixing is neglected, i.e., J = 1 in Eq (10). If only transitions from the vibrational ground state (μ = 0) are considered, i.e., for the 0→υ vibrational transition, the temperature-averaged FCFs turn into the standard Poisson distribution [32,55]:

$${FCF}_{\left(\mu =0,\upsilon \right)}=exp(-\bar{S})\frac{{\bar{S}}^{\upsilon }}{\upsilon !}$$ (15)

In this case, the molecular spectrum can be modeled as a FC progression [32,55,56], and the relative intensity of a multidimensional vibrational transition is obtained as a simple product of one-dimensional FC integrals [32]:

$${I(\omega )}_{\left(\mu =0,\upsilon \right)}\propto \sum_{\upsilon_{j}}exp(-{\bar{S}}_{j})\frac{{\bar{S}}_{j}^{\upsilon_{j}}}{\upsilon_{j}}\Gamma (\hbar \omega -E_{0}+\upsilon_{j}\hbar \omega_{j})$$ (16)

where *Γ*(*ћ*ω-E_0_+υ_j_*ћ*ω) is the line shape function, which we assume to be Gaussian with constant width; υ_j_ is the number of phonons of mode ω_j_ in the final vibrational state; and E_0_ is the 0→0 transition energy.

The overall line shape is assumed as a convolution of the intensity distribution function with the δ-function replaced by a line shape function *Γ*_N_(ω) [57,58]. Jortner [59] proposed that for the ET rate expression, the contribution of the solvent modes was lumped into a Gaussian line shape. In this study, the calculated spectral intensities are a set of vertical lines underlying vibrational modes (abscissa). Following Turin’s suggestion [11] that the resolution of human olfactory spectrometers is approximately 400 cm^-1^ for the width of inelastic electron tunneling spectroscopy (IETS), we applied a Gaussian function with a standard deviation of 100 cm^−1^ to convolute the vertical lines. In this way, the simulated spectrum became a smooth curve with characteristic of width and intensity, which was similar to the experimental spectrum, thus making it convenient to compare different molecules.

## Results

### Spectral comparison of HCN, benzaldehyde and nitrobenzene

The results of Huang-Rhys factors $\bar{S}$ for HCN, benzaldehyde and nitrobenzene are listed in Table 1, S1 and S2 Tables. To demonstrate the accuracy of the B3LYP/6-31G method used for the frequencies and $\bar{S}$ factors, the calculated IR spectra were compared with the measured IR spectra for HCN, benzaldehyde and nitrobenzene. As expected, all major experimentally resolved IR active vibrations are correctly described in the calculation; the calculated and measured vibrational frequencies show almost perfect agreement. The performed comparison clearly demonstrates that B3LYP/6-31G is a reliable method for the calculation of vibrational spectra; it is therefore employed in the further analysis. More detailed information is provided in the supplementary information (S1 Text and S1 Fig).

**Table 1:** The Huang-Rhys Factors $\bar{S}$ and intramolecular reorganization energies λ_i_ (eV) for each vibrational frequency ω_i_ (cm^-1^) of HCN in its neutral and anionic states.

	Neutral			Anionic		
ω_i_	$\bar{S}$	λ_i_	ω_i_	$\bar{S}$	λ_i_	vibrational mode
778	0	0	696	0	0	bend
2149	1.978	0.527	1704	1.594	0.337	C≡N stretch
3500	0.084	0.036	3446	0.029	0.013	C-H stretch

When a tunneling electron is added, the electronic state of HCN is changed from a neutral state to an anionic state, the spin population is primarily distributed at the C≡N bond (S3 Table), the geometry changes correspond to an elongation of these three bonds, which lead to the effect on intramolecular reorganization energy, the largest at 2149 cm^‒1^ in the neutral state and 1704 cm^-1^ in the anionic state, and the corresponding Huang-Rhys factors $\bar{S}$ = 1.978 and 1.594, respectively. During the vibronic transition, the HCN bending and C-H bond length will be more or less unchanged, their Huang-Rhys factors $\bar{S}$ = 0 and 0.084 are in the neutral state, and $\bar{S}$ = 0 and 0.029 in the anionic state (Table 1).

**Fig 2:**
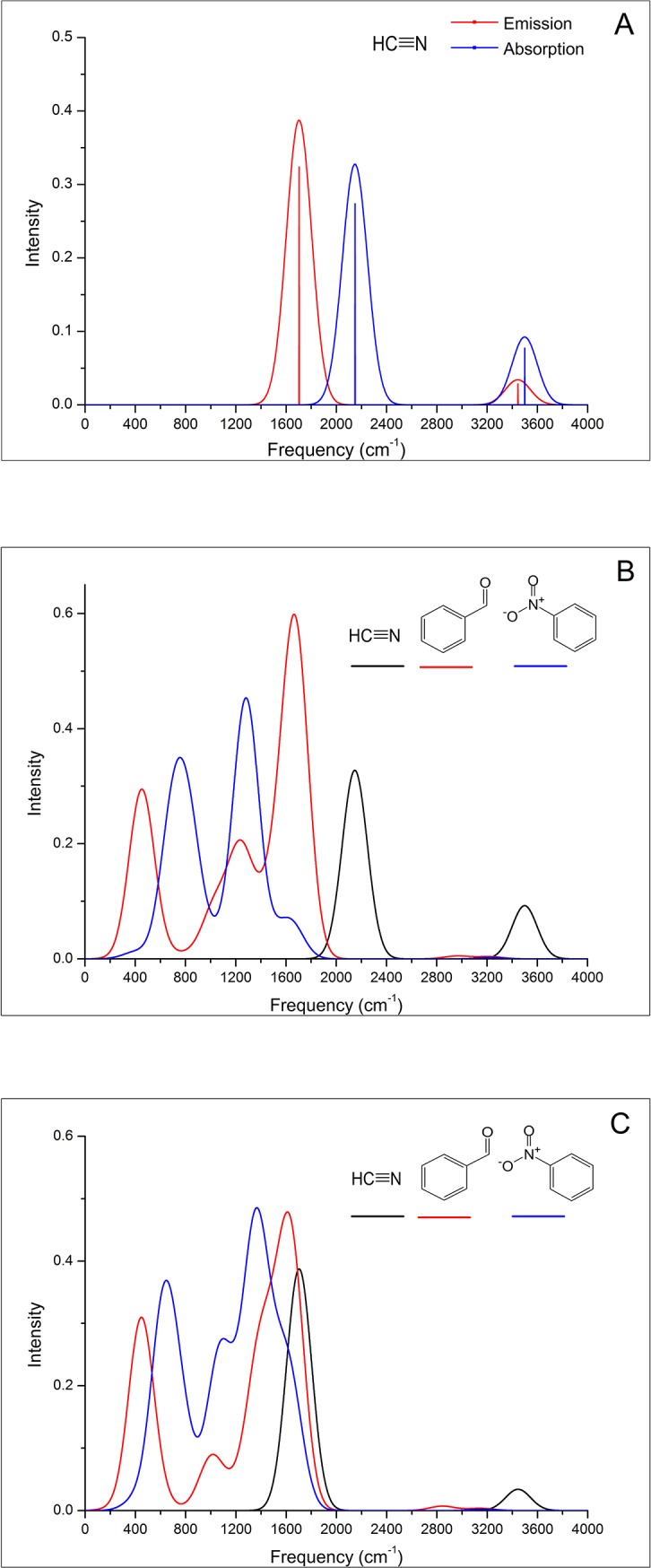
**A.** The structure and ET absorption and emission spectra of HCN. The calculated peaks are shown before (vertical bars) and after (smooth curve) convolution with a Gaussian (s.d. = 100 wavenumbers). **B.** The structures and ET absorption spectra of HCN, benzaldehyde and nitrobenzene. **C.** The structures and ET emission spectra of HCN, benzaldehyde and nitrobenzene.

According to Eq (1), we can simulate the ET spectra with the Huang-Rhys factors ${\bar{S}}_{j}$ and the final vibrational quantum numbers υ_j_. For the ET reaction in the mixed quantum classical regime, υ_j_ depends on the magnitude of the ${\bar{S}}_{j}$. Jortner [60] has reported that for an ET process at high temperature the activation energy exhibits a parabolic dependence, $E_{A}=\hbar \omega {\left(\upsilon_{j}-\bar{S}\right)}^{2}/4\overset{-}{S}$, for activationless processes υ_j_ = $\bar{S}$. Therefore, based on the Huang-Rhys factors $\bar{S}$ = 1.978 and 1.594 for HCN, we speculated that the vibronic transition excites two phonons in this odorant. Fig 2A shows that the calculated ET absorption and emission spectra of HCN before (vertical lines) and after (smooth curve) convolution; the emission band (approximately 1704 cm^-1^) is left-shifted compared with the absorption band (approximately 2149 cm^-1^).

It should be noted that the ET emission spectra are not the vibrational spectra of the negatively charged states, similarly, ET absorption spectra are not the vibrational spectra of neutral states, because ET spectra origin from vibronic transition and are calculated from Huang-Rhys Factors $\bar{S}$. For HCN, the intensity value of the ET spectrum of HCN is not directly related to the IR intensity of the corresponding vibrations, as illustrated in Fig 2A and S1A Fig, the C-H stretching mode and bending mode have the large IR intensity, but the intensity of the ET spectrum is very low. For the C≡N stretching mode, the case is the opposite. The reason can be explained as follows: for the IR of HCN in the ground state, the C≡N stretching does not produce a change in the dipole moment, and the moment remains zero throughout the vibration by symmetry, but the C-H stretching mode and bending mode can produce a change in the dipole moment during the vibration, leading to IR activity.

Fig 2B and Fig 2C show that both ET absorption and emission spectra of benzaldehyde are composed of two prominent peaks at approximately 450 cm^-1^ and 1600 cm^-1^, and a lower, smaller peak at 1000 cm^-1^. The first peak in the low-frequency region of the spectra is dominated by a vibronic progression originating from the coupling to vibrational mode with bending vibration in-plane at a calculated 453 cm^-1^ in the neutral state and 448 cm^-1^ in the anionic state. The third prominent peak in the high-frequency region of the spectra is dominated by vibronic progression originating from the coupling vibrational modes are the aromatic C = C stretch as well as C = O stretch at 1660 cm^-1^ and 1707 cm^-1^ in the neutral state and at 1578 cm^-1^ and 1654 cm^-1^ in the anionic state (S1 Table).

Fig 2B and 2C also show that both the ET absorption and emission spectra of nitrobenzene are composed of two peaks. For the first peak of the absorption spectrum, the obvious vibronic feature corresponding to the vibrational mode with benzene ring bending vibration in-plane is at a calculated 699 cm^-1^, the nitro group scissoring vibration is at a calculated 831 cm^-1^ in the neutral state. For the first peak of the emission spectrum, the coupling vibrational modes are calculated at 626 cm^-1^ and 772 cm^-1^ in the anionic state. The second peak of the absorption spectrum, which is in the high-frequency region and is primarily dominated by vibronic progression originating from the coupling to vibrational mode with the benzene ring C-H rocking vibration in-plane and the N = O stretch in the nitro group, is calculated at 1290 cm^-1^. For the second peak of the emission spectrum, the coupling vibrational modes are calculated at 1069 cm^-1^, 1359 cm^-1^, 1524 cm^-1^ and 1641 cm^-1^ in the anionic state, respectively (S2 Table).

Quantitative spectral comparison could be used to investigate which of the two types of ET spectrum (absorption and emission) could be the odorant characteristic spectrum. As shown in Fig 2B, comparing the calculated ET absorption spectrum of HCN with that of benzaldehyde and nitrobenzene using an overlap criterion, the prominent peak at approximately 2149 cm^-1^ of HCN has no overlap with the peaks of the other two odorants. In contrast, compared with the emission spectra, as shown in Fig 2C, the prominent peak of HCN at approximately 1704 cm^-1^ has 90% band overlap with the third peak of the benzaldehyde, nitrobenzene also gives a good fit to the HCN band, the second peak of nitrobenzene considerably overlaps with the third peak of benzaldehyde.

Remarkably, for the third peak of the emission spectra of benzaldehyde, the coupling vibrational modes are the aromatic C = C stretch as well as C = O stretch at 1578 cm^-1^ and 1654 cm^-1^ in the anionic state, which involve the odotope (C = C–C = O) identifies by Zakarya et al. [61] as responsible for the odor of bitter almonds. We assume that the molecules with similar odors should have similar spectra, the common spectral feature for odorants correlates with a certain odor character. Because the three structurally different bitter-almond molecules have a common spectral feature at approximately 1600 cm^-1^ in the emission spectra, we deduced that the emission spectrum of ET can act as the odorant characteristic spectrum.

To verify the hypothesis that the ET emission spectrum can act as the odorant characteristic spectrum, a good way would be to compare the spectra of two or more molecules with very similar odor characters and very different structures [11]. In the present study, we add two sets of examples: (i) two chemically unrelated odorants, namely, karanal (2-(2,4-dimethyl-3-cyclohexen-1-yl)-5-methyl-5-(1-methylpropyl)-3-dioxane, C_17_H_30_O_2_) and cedramber (1H-3a,7-Methanoazulene,octahydro-6-methoxy-3,6,8,8-tetramethyl-, (3R,3aS,6R,7R,8aS)-, C_16_H_28_O), both of which have ambergris odors [11], with karanal being slightly greener and cedramber being somewhat woodier; and (ii) two chemically unrelated odorants, namely, 1,7,7-trimethylbicyclo[2.2.1]heptan-2-one (C_10_H_16_O) and ethylphosphoramidothioic dichloride (C_2_H_6_NPSCl_2_), both of which have camphoraceous odors [5].

We calculated the ET emission spectrum for each molecule mentioned here with the same methods, i.e., using the B3LYP/6-31G method to perform geometry optimizations and using the DUSHIN program to calculate the Duschinsky rotation matrix. As shown in S2 Fig, the calculated ET emission spectra of cedramber and karanal are highly overlapped at approximately 400 ± 200 cm^-1^; similarly, the calculated ET emission spectra of 1,7,7-trimethylbicyclo[2.2.1] heptan-2-one and ethylphosphoramidothioic dichloride are highly overlapped at approximately 600 ± 200 cm^-1^, consistent with their similar smells.

We expanded the scope of test examples and chose a broad set of odorants belonging to widely different structural and odor classes, such as musks, woods, violets, and some of the other bitter almonds. The spectral comparison results of these odorants are consistent with the spectral comparison of the small subset (HCN, benzaldehyde and nitrobenzene). For odorants with similar odors, the common spectral feature can be found in the emission spectra, no matter whether their structures are the same, while in the absorption spectra, we did not find any common features.

Some remaining discrepancy could be due to Duschinsky mixing, because we adopt the DUSHIN program[42] to calculate the normal-mode-projected displacements and Duschinsky rotation matrices with the use of curvilinear coordinates, preliminary calculations of Duschinsky matrices point to the presence of some Duschinsky mixing among the vibrations in benzaldehyde and nitrobenzene (S4 and S5 Tables). Additionally, to allow large-amplitude curvilinear motions to be partitioned into individual vibrational components using the machinery of normal-mode analysis, the DUSHIN program ignores the coordinate dependence of the Jacobian describing the curvilinear-coordinate transform and hence ignores all kinetic energy anharmonicity effects. To further improve the spectral similarity for molecules with the same odor, investigations are currently in progress.

### Isotope effect

A classical approach to test the plausibility of the vibrational theory is to compare the odor character difference between the deuterated isotopomers. However, some psychological experiments can hardly be interpreted by the previous vibrational theories. Keller and Vosshall [14] reported that naive subjects are incapable of distinguishing acetophenone and acetophenone-d8. Gane et al.[21] confirmed their results on trained subjects. In the same article, Gane et al. additionally reported that trained subjects can easily distinguish deuterated and undeuterated musk odorants, cyclopentadecanone and cyclopentadecanone-d28.

**Fig 3:**
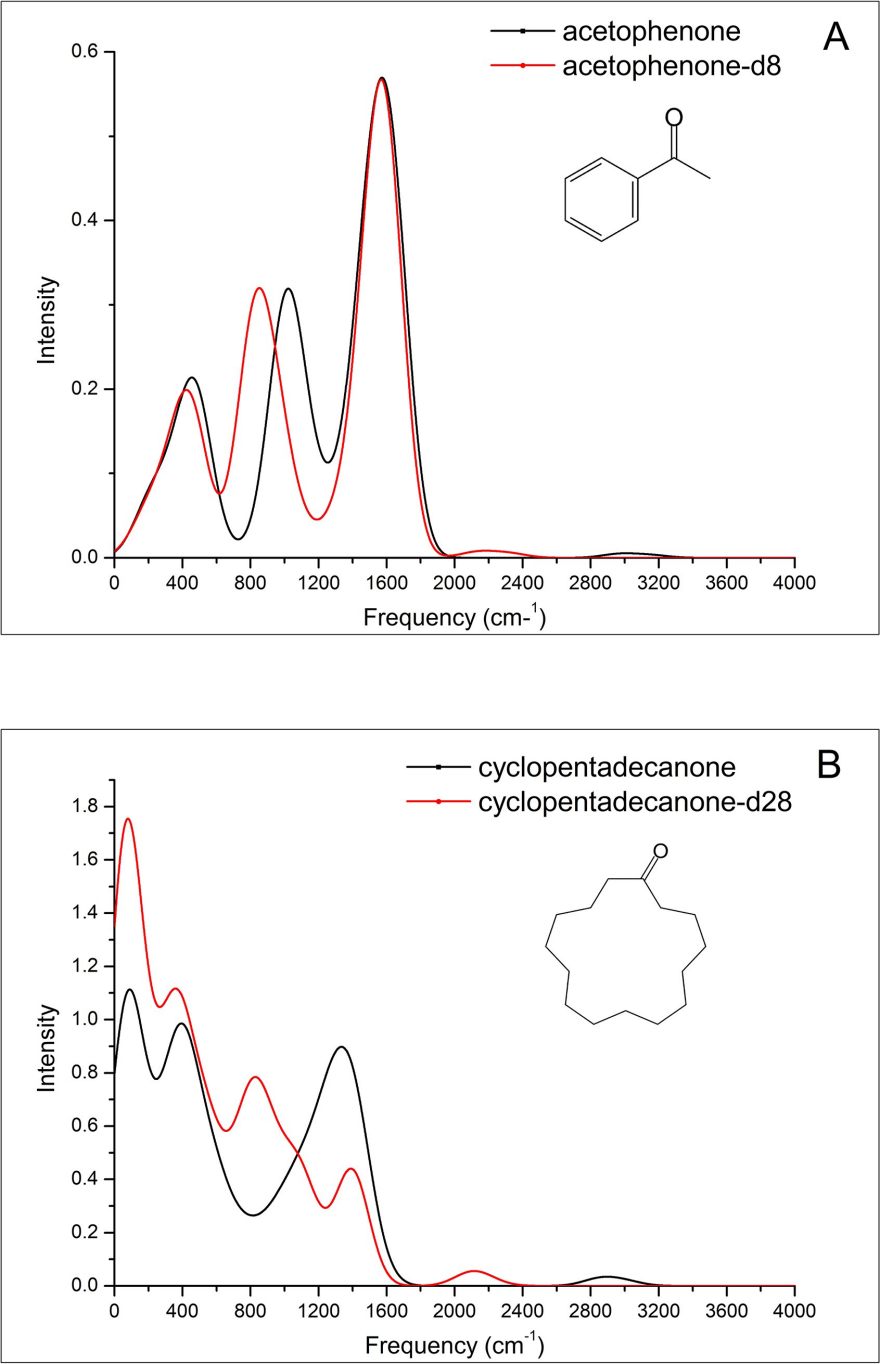
**A.** The structures of acetophenone and ET emission spectra of acetophenone and acetophenone-d8. **B.** The structures of cyclopentadecanone and ET emission spectra of cyclopentadecanone and cyclopentadecanone-d28.

In this study, we attempt to interpret the controversial phenomenon of the experimental results by ET spectroscopic analysis. As shown in Fig 3A, both emission spectra of acetophenone and acetophenone-d8 have three peaks after convolution. For the first peak at approximately 400 cm^-1^ in the low-frequency region, both spectra largely consist of a series of vibronic features corresponding to the molecular skeleton vibration. The obvious vibronic feature in non-deuterated corresponds to vibrational mode at a calculated 480 cm^-1^ with CH_3_-CO-C_6_H_5_ scissoring vibration, as well as benzene ring breathing vibration in the anionic state, with a Huang-Rhys factor of $\bar{S}$ = 0.177; for deuterated acetophenone, the corresponding mode is calculated at 450 cm^-1^ with a Huang-Rhys factor of $\bar{S}$ = 0.159 (S6 and S7 Tables). For the second peak, the obvious vibronic feature for non-deuterated acetophenone corresponding to the vibrational mode with benzene ring breathing vibration and sp^3^ C-H deformation vibration is calculated at 1014 cm^-1^, with a Huang-Rhys factor of $\bar{S}$ = 0.188; for deuterated acetophenone, the corresponding mode is calculated at 811 cm^-1^ with a Huang-Rhys factor of $\bar{S}$ = 0.21. For the third peak, the emission spectra largely consist of a series of vibronic features corresponding to the benzene ring C-H anti-symmetric stretching vibration approximately 1630 cm^-1^ for both non-deuterated and deuterated acetophenone.

By spectroscopic analysis, we can see that the first and third peak of the two isotopes are overlapping at approximately 400 cm^-1^ and 1630 cm^-1^; for the second peak, both the shape and the intensity of the two isotopes are almost identical, but the position of the deuterated peak showed a left-shift of approximately 200 cm^-1^ compared to the non-deuterated peak. According to Turin’s theory [11], human olfactory receptors constitute biological spectrometers with poor resolution (~400 cm^-1^), it is reasonable to say that ordinary human subjects cannot detect the effect of deuterium on odor character in acetophenone. Nevertheless, the odor character difference between acetophenone isotopomers, which are associated with the slightly different locations of the second peaks of ET emission spectra, could be detected by some animals with keen olfactory sense. For instance, Franco et al.[16] has reported that flies can use olfaction alone to discriminate isotopic acetophenone odorants.

As presented in Fig 3B, cyclopentadecanone has a macrocyclic ketone structure with a 15-membered ring. For its deuterated and undeuterated isotopomers, each molecule has more than 120 vibrational modes (S8 and S9 Tables). The intensities of the vibronic emission spectrum in each vibrational mode may not be large, but they can be combined to form a large peak after convolution. The spectrum of non-deuterated cyclopentadecanone has 3 peaks within 2000 cm^-1^, while the spectrum of cyclopentadecanone-d28 has 4 peaks. The first and second peak of the two isotopes are at the same position, while the intensity of deuterated cyclopentadecanone is greater than that of non-deuterated cyclopentadecanone. Given the emission spectra of the two isotopic cyclopentadecanone odorants are completely different, their odor differences should be easily perceptible.

To interpret deuteration can alter the odor character of cyclopentadecanone but not that of acetophenone, Gane et al.[21] proposed that deuteration exerts the largest effect on the parts of the vibrational spectrum involving C-H motions, odor character might be detectable in odorants containing more C-H groups, since the C-H bond is weakly polar, a bond of low polarity may be difficult to detect by smell. In contrast to acetophenone which contains only 8 carbons and 8 hydrogens, cyclopentadecanone has 15 carbons and 28 hydrogens, more than 3 times the number of vibrational modes involving hydrogens than in acetophenone. This explanation does not satisfy the opponents of the vibrational theory. Hettinger [17] stated that the comparison between C-H with C-D stretching modes showed an obvious isotope shifts from the 3000 cm^-1^ region into the 2200 cm^-1^ region, the difference is as great as half of the entire spectral range in human vision or half an octave in hearing. Accordingly, the odor character difference between acetophenone isotopomers should be easy to detect; however, this is not the case.

As shown in Fig 3A and 3B, there's a very low, flat peak at the 3000 cm^-1^ for non-deuterated acetophenone and cyclopentadecanone, the corresponding modes are C-H stretch modes in the anionic state; and a similar low, flat peak at the 2200 cm^-1^ region for deuterated acetophenone and cyclopentadecanone, the corresponding modes are C-D stretch modes in the anionic state. By spectroscopic analysis, we conclude that C-H and C-D stretch modes have little effect on the switch from the anionic state to the neutral state geometries of acetophenone and cyclopentadecanone, and have little to do with odor recognition, despite the significant change in C-H stretch frequency upon substitution by deuterium. Both C-H and C-D stretch modes have the large IR intensity, but the corresponding intensity of the ET emission spectra are very low, this proved once again that ET emission spectra are not the vibrational spectra of the negatively charged states.

## Discussion

Charge transfer (CT) is ubiquitous in biology [16–21]. There are two types of CT: (1) The hole transfer (HT) in the case of the radical cations, and (2) The electron transfer (ET) in the case of the radical anions. The type of CT may occur via two different mechanisms: the super-exchange (coherent) transfer or the sequential (hopping) transfer [62–67]. Correspondingly, we think placing an odorant molecule (B) between the D and A molecules allows the ET to occur either as a single coherent scattering event from D to A directly (super-exchange regime), or as a sequence of two incoherent hops, first from D to B then from B to A (sequential hopping regime).

Turin [11] proposed that Inelastic electron tunneling spectroscopy (IETS, defined as the second derivative of the tunneling current to the bias voltage) can be as a model for the olfactory recognition mechanism. Hoehn et al. [24] extended this hypothesis to GPCRs within the mammalian nervous system, IETS has been proposed as a model for the mechanism by which GPCRs are activated by a bound agonist. The IETS model indicates a superexchange tunneling regime [68–72], in which the tunneling electron does not pass through the molecule between the electron source (D) and sink (A). The molecule remains in a neutral state throughout the process of ET, which means that the signal is driven only by the vibrational modes of the odorant and not by its ability to transfer an electron[29]. Therefore, according to the theory developed by Sleigh et al.[73], the IET spectrum intensity for a given active vibrational mode j can be approximated by:

$$I_{j}=\sum_{i=1}^{N}I_{i,j}=\sum_{i=1}^{N}q_{i}^{2}{\left(\Delta x_{i,j}\right)}^{2}$$ (17)

where the sum is over all atoms within the molecule, q_i_ is the partial charge of atom i, and Δx_i,j_ is the Cartesian displacement of atom i in mode j.

In order to investigate which ET regime is the dominated mechanism, we referred to the experimental results described by Paulson et al.[74]. They performed a series of experiments to determine the rate of intramolecular charge transfer, finding that the free energy of formation ($\Delta G_{I}^{0}$) of the intermediate state was the critical variable. Specifically, the super-exchange is the dominant mechanism when $\Delta G_{I}^{0}$ is large (2 eV), while the sequential mechanism will dominate when $\Delta G_{I}^{0}$ is small (0.5 eV). In our DBA model, we have three relevant diabatic states denoted by D^-^BA, DB^-^A, and DBA^-^ corresponding to the initial, intermediate, and final electronic states of the system. According to Jortner's theory [59], to undergo ET with energy conservation in the mixed quantum classical regime, the energy difference between D^-^BA and DB^-^A states, $\Delta G_{I}^{0}$, should be comparable to the sum of the reorganization energy of the environment λ_S_ and the initial product vibrational energy (υħω), otherwise the ET process becomes suppressed. Despite λ_S_ being unknown experimentally for the OR, we know that for primary ET processes in the protein photosynthetic reaction centers λs ≈ 500–3000 cm^-1^ [75]. Thus, $\Delta G_{I}^{0}$ values are in the range of 10^−3^ eV–1 eV. In combination with the experimental evidences [74], we speculated that the sequential (hopping) regime is the dominated ET mechanism for olfaction.

In the present study, we revealed that the emission spectrum of ET could act as an objective evaluation criterion for odor character based on the sequential ET regime. Comparing the ET emission spectrum with the IET spectrum, we analyze specific examples: (i) Turin[13] argued that bitter almonds was a ‘bichromatic’ odor based on IET spectra; the most prominent peak for 8 bitter almond odorants in the region below 1500 cm^-1^ lies near 920 cm^-1^ and matches the HCN bend vibrational mode. In our study, we found that in the ET emission spectra of three molecules (HCN, benzaldehyde, and nitrobenzene), the overlapping peaks at approximately 1600 cm^-1^ match the HCN C≡N stretch vibrational mode (Fig 2C). (ii) The IET spectra of nondeuterated acetophenone and acetophenone-d8 showed that the peak corresponding to the C-H wag modes at ~1400 cm^-1^ is absent in acetophenone-d8 and that the C-H stretches are shifted into the region (~2200 cm^-1^), suggesting that odor differences should be perceptible; While the ET emission spectra showed that the isotopologues of acetophenone have extremely similar spectra (Fig 3A), suggesting that odor differences should not be perceptible by human subjects.

Turin [11] proposed that the OR function can act as a tunneling spectroscope, unlike the typical experimental IETS procedure, 'biological IETS' does not involve scanning of the entire vibrational range (from 1 to 4000 cm^-1^); instead, the range of vibrational energies is covered piecewise by a series of receptors tuned to different energies, that is, to cover the vibrational spectrum of the entire vibrational range, several receptor classes would be required, each tuned to a different segment of the vibrational spectrum. Hoehn et al.[24] proposed that agonists of a particular protein would share a single spectral feature associated with the inelastic electron transfer. The IET spectra of several selected 5-HT_2A_ agonists were modeled using quantum chemical method, they found that all agonists are capable of facilitating electron transfer within the same energy region around the 1500 ± 35 cm^-1^.

For two odorants that possess similar odor characteristics but have different structures, such as HCN and benzaldehyde, we investigated their emission spectrum of ET and found that these two molecules have a common spectral feature, with overlapping peaks at approximately 1600 cm^-1^. According to vibrational theory, HCN and benzaldehyde should be capable of facilitating electron transfer within the same energy region around 1600 cm^-1^. Therefore, these two molecules will dock into a set of detecting sensors (the ORs), which can detect the molecule vibration frequency around the 1600 cm^-1^ region and activate these ORs, causing the protein to release a G-protein.

Hoehn et al.[24] calculated the integral of the tunneling probability density around the 1500 cm^-1^ region after modeling the IET spectra of several selected 5-HT_2A_ agonists. The integrals are found to be in good qualitative agreement with the inverse EC50 for each 5-HT_2A_ agonist. Some agonists (two PIAs, DOI and DOB) possess great differences in their potency [76] because they have different tunneling probability densities around the 1500 cm^-1^ region, although they have similar docking affinities at the 5-HT_2A_ receptor [77].

The study by Hoehn et al.[24] indicated that the activation intensity of the receptor is mainly determined by the probability of electron transfer, not just by the docking affinity. By investigating the emission spectrum of ET, we found that HCN and benzaldehyde have similar intensities at 1600 cm^-1^, which indicates that these two molecules’ vibronic transition probabilities from the anionic state to the neutral state are similar, that is, the probabilities of electron transfer from the odorant (B) to the acceptor molecule (A) are similar. We think that when HCN and benzaldehyde dock into the binding pocket within the same OR, although their binding energies are different, the protein responses should be similar due to the similar electron transfer possibilities.

The positively charged molecules may be more stable than negatively molecules, Bittner et al.[18] suggested that the charge transfer type of the DBA model is the hole transfer, and the odorant molecule undergoes electronic change from the neutral state to the cationic state. However, Pshenichnyuk et al.[78] proves the electron-accepting properties of odorant molecules are involved in their smell recognition with experiments. Their study is based on the theory which is known as dissociative electron attachment (DEA) mechanism [79,80], using DEA spectra as odor characteristic spectra can interpret a series of mustard oil odorants structurally close odorants with markedly different odor characters.

**Fig 4:**
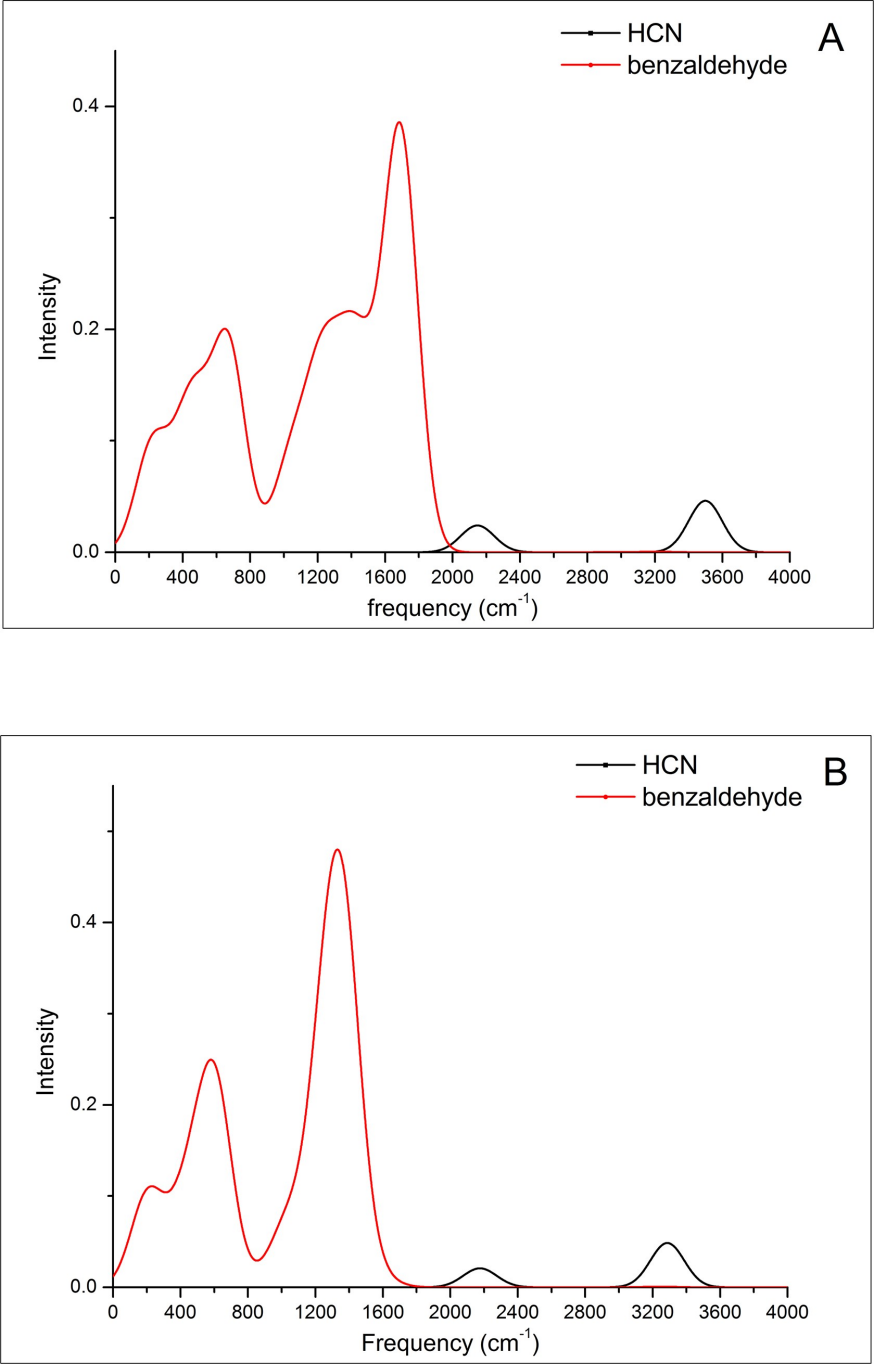
Comparative spectral analysis in hole transfer way for HCN and benzaldehyde. **A.** the ET absorption spectra. **B.** the ET emission spectra.

Our DBA model agrees with the DEA mechanism in terms of resonance electron attachment with formation of the temporary negative ion and its decay by electron detachment, but it should be noted that the DEA mechanism involves an irreversible chemical reaction, i.e., dissociation of the odorant anion. We assume that most of the negatively charged odorants do not dissociate at room temperature in the air, Brookes [81] stated that olfaction does not alter the chemical composition of the odorant. To investigate whether hole transfer or electron transfer dominates the signal transduction in the OR, we also calculated hole transfer spectra for each molecule mentioned in this article with the B3LYP/6-31G method. For example, as shown in Fig 4, the calculated hole transfer spectrum of HCN has no overlap with that of benzaldehyde. We conclude that neither hole transfer absorption spectra nor emission spectra can serve as a measure of odor character.

A convenient way to test the vibrational theory is the experimental verifications of the isotope effect, because replacing hydrogen with deuterium (mass change from 1.0078 to 2.0141) in an odorant exerts the largest effect on the parts of the vibrational spectrum, while the chemical effects are small. Behavioral experiments showed that flies [16,27], honeybees [22,26] and humans [21] can distinguish the isotopologues of odorants. At the receptor level, the study by Block et al.[23] suggests that ORs are unable to distinguish between the isotopologues of odorants. Hoehn et al.[28] used a pair of agonists (both deuterated and nondeuterated) of the 5-HT_2_ class of serotonin receptors to measure the receptor affinity and receptor activation with the calcium flux approach. The results indicated that there was no evidence to support the claim that selective deuteration affected either the binding affinity or the activation by the selected ligands for the examined members of the 5-HT_2_ receptor class.

We think that these receptor experiments prove only that it is impossible to recognize deuteration by relying on some overly specific receptors. Note that one odorant can activate numerous types of ORs, while a single OR can be activated by several different odorants [82]. Turin et al.[83] supposed that it cannot exclude the possibility that other ORs are weakly activated by cyclopentadecanone or its deuterated isotopomer. Sell [84] proposed that an odorant activating a given receptor does not enable prediction of the ultimate odor percept. Triller et al.[85] investigated the response of the human olfactory receptor OR1D2 to a broad array of muguet odorants. They observed that OR1D2 activation does not correlate directly with any specific odor percept and that some molecules that fail to activate OR1D2 can also elicit the muguet character. In addition, Block et al. used a heterologous functional expression for ORs in human embryonic kidney 293 (HEK293) cells to study the response of ORs, which lack the in situ environment [83].

Recently, some in vivo studies showed that an insect’s olfactory system can discriminate between isotopomers of the same odorant [26,27], which provided experimental evidence for vibration theory. Paoli et al.[26] examined the local activity of specific odorants at the glomeruli of the honey bee (Apis mellifera). Odor response patterns were measured in a subset of 19 glomeruli by monitoring the intracellular calcium concentration in the presence of controlled olfactory stimulation, which was followed by employing two-photon functional microscopy to assist in visualizing the local activity on the topography of the sensory organ. Their results show that several of the glomeruli displayed differential flux responses with respect to exposure to isotopologues of an odorant. Specific glomeruli in the same animal were preferentially activated by one of the isotopologues, while others exhibited the same response to both isotopologues, and some were inhibited by one of a pair of isotopologues. The responses of these specific glomeruli indicate that deuterated and nondeuterated odorants can generate different neuronal activation maps, indicating that bee ORs can discriminate between isotopomers of the same odorant.

Drimyl et al.[27] assessed the physiological response of Drosophila antennae to multiple odorant isotopologues using electroantennograms (EAGs) and behavioral approaches. By analyzing the shape and magnitude of each EAG, they found that both isotopologues can activate common ORs but do not activate the common ORs equally, which is reflected in the EAG shape similarity and amplitude differences. In addition, they found that each isotopologue can also activate additional unique ORs. Maybe someone will think that the insect ORs are ionotropic receptors (IRs), while vertebrate ORs are GPCRs, each class of receptors shows specific evolutionary benefits [86,87]. To rigorously verify the isotope effect presented by the vibrational theory of olfaction, we suggest that it is better to use mice or other vertebrate models as test subjects to study the response to multiple odorant isotopologues with physiological and behavioral approaches.

Block et al.[23] also asserted that there are no experimental data showing direct evidence of electron transfer, or the effect of odorant vibrations being responsible for triggering ORs response. They further claimed that no evidence exists that GPCRs require electron transfer for their activation. One major objection to this statement is the existence of the case of the rhodopsin, a photoreceptor belonging to the GPCR class, a chromophore called 11-cis-retinal that binds to the rhodopsin via a Schiff base (RSB) and the ε-amino group of a lysine side chain in the middle of TM7 [88]. In the ground state, the 11-cis-retinal is positively charged because the retinal RSB is protonated (RSBH^+^). When photoexcitation occurs, the bond of the retinal is altered, charge transfer occurs, and the positive charge is displaced from the RSBH+ to the β-ionone ring, leading to a neutralization of the RSBH^+^ [88–91].

From the point of view of molecular interaction, if the two molecules are close enough to interact with each other, there is a charge transfer energy component in the total intermolecular interaction energy [92–94]. By the energy decomposition analysis (EDA), the intermolecular interaction energy can be partitioned into energy components such as electrostatic, polarization, charge transfer, exchange and correlation contributions and related chemical phenomena [95]; here, charge transfer refers to interactions between the occupied orbital of the donor molecule and unoccupied orbital of the acceptor molecule, and the orbital energy gap and overlap are important factors. One quantum mechanics approach, Fragment Molecular Orbital (FMO), provides accurate information on the individual contribution of each residue of protein to the ligand-binding energy [96]. Heifetz et al.[97] have applied the FMO method to 18 Class A GPCR−ligand crystal structures; they observed that the average charge transfer fraction contribution for the 18 crystal structures is up to 17%.

If our DBA model of vibrational theory is validated, it will be possible to predict the odor perception of a molecule simply from spectroscopic analysis, without relying on trial-and-error and the aggregation of opinions gathered from panels of humans, which can dramatically accelerate the development of novel ingredients by the fragrance industry.

The core of vibration theory is that the ORs are activated through an ET mechanism; however, evaluating the reliability of the ET mechanism of olfaction cannot be addressed until the complete structure of the OR is known; perhaps, the most important development will be the production of a high-quality X-ray structure of an OR.

## Supporting information

S1 Text Spectral comparison of HCN, benzaldehyde and nitrobenzene. (DOCX)

S1 Fig The IR spectra for HCN, benzaldehyde, and nitrobenzene. A, HCN; B, benzaldehyde; and C, nitrobenzene.
(PDF)

S2 Fig The structures of two ambergris odorants, two camphoraceous odorants, and their ET emission spectra. **A. The structures of compound 1,** karanal (2-(2,4-dimethyl-3-cyclohexen-1-yl)-5-methyl-5-(1-methylpropyl)-3-dioxane, C_17_H_30_O_2_); **compound 2,** cedramber (1H-3a,7-Methanoazulene,octahydro-6-methoxy-3,6,8,8-tetramethyl-, (3R,3aS,6R,7R,8aS)-, C_16_H_28_O); **compound 3,** 1,7,7-trimethylbicyclo[2.2.1]heptan-2-one (C_10_H_16_O) and
**compound 4,** ethylphosphoramidothioic dichloride (C_2_H_6_NPSCl_2_).
**B. The ET emission spectra of karanal and cedramber**.
**C. The ET emission spectra of 1,7,7-trimethylbicyclo[2.2.1]heptan-2-one and ethylphosphoramidothioic dichloride**.
(PDF)

S1 TableThe Huang-Rhys Factors, $\bar{S}$ and intramolecular reorganization energies, λ_i_ (eV) for each vibrational frequency, ω_i_ (cm^-1^) of benzaldehyde in its neutral and anionic states.(DOCX)

S2 TableThe Huang-Rhys Factors, $\bar{S}$ and intramolecular reorganization energies, λ_i_ (eV) for each vibrational frequency, ω_i_ (cm^-1^) of nitrobenzene in its neutral and anionic states.(DOCX)

S3 Table The spin population of HCN when a tunneling electron is added. (DOCX)

S4 Table Duschinsky mix for selected vibrations of bezaldehyde. (DOCX)

S5 Table Duschinsky mix for selected vibrations of nitrobenzene. (DOCX)

S6 TableThe Huang-Rhys Factors, $\bar{S}$ and intramolecular reorganization energies, λ_i_ (eV) for each vibrational frequency, ω_i_ (cm^-1^) of acetophenone in its neutral and anionic states.(DOCX)

S7 TableThe Huang-Rhys Factors, $\bar{S}$ and intramolecular reorganization energies, λ_i_ (eV) for each vibrational frequency, ω_i_ (cm^-1^) of acetophenone-d8 in its neutral and anionic states.(DOCX)

S8 TableThe Huang-Rhys Factors, $\bar{S}$ and intramolecular reorganization energies, λ_i_ (eV) for each vibrational frequency, ω_i_ (cm^-1^) of cyclopentadecanone in its neutral and anionic states.(DOCX)

S9 TableThe Huang-Rhys Factors, $\bar{S}$ and intramolecular reorganization energies, λ_i_ (eV) for each vibrational frequency, ω_i_ (cm^-1^) of cyclopentadecanone-d28 in its neutral and anionic states.(DOCX)
