# Supplementary material for: Exploring the mechanism of olfactory recognition in the initial stage by modeling the emission spectrum of electron transfer
Source: PLoS One. 2020 Jan 10;15(1):e0217665. doi: 10.1371/journal.pone.0217665 (PMC6953861; doi:10.1371/journal.pone.0217665)
Supplement: S1 Text — (DOCX) [file pone.0217665.s001.docx]

**Spectral Comparison of HCN, Benzaldehyde and Nitrobenzene.**

The vibrational states of a molecule can be probed using various means, and the most direct approach is through infrared (IR) spectroscopy. Resolution is one of the measurement parameters involved in obtaining IR spectra. With general-purpose infrared spectrophotometers, the selected resolution is usually within the range of 16 cm^-1^ to 0.5 cm^-1^. In the present study, we chose the calculated IR spectra of three odor molecules (HCN, benzaldehyde and nitrobenzene) to compare with the experimental IR data. According to the literature [[1-5](#_ENREF_1)], we know that these IR spectra of three odor molecules can be obtained from samples in all phases (liquid, solid and gaseous). To make it convenient to compare the calculated spectra with the experimental IR data, we only chose the experimental IR data obtained from the samples in a liquid state. For molecules in the liquid state or in solution, collision and proximity broadening predominate, and lines are much broader than lines from the same molecule in the gas phase[[6](#_ENREF_6)]. Line maxima may also be shifted. Because there are many sources of broadening, the lines have a stable distribution, tending towards a Gaussian shape. For this reason, the resolution is usually set to approximately 4 cm^-1^ for solid and liquid samples. Therefore, we use a broadening factor of 4 cm^-1^ and compiled for presentation using Excel.

The IR spectrum of HCN has been measured experimentally [[1](#_ENREF_1)]. It has only three vibrational modes, a bending mode at 712 cm^−1^ (ν2), C-H stretch at 3289 cm^−1^ (ν3) and C≡N stretch at 2100 cm^−1^ (ν1); the mode of the C≡N stretch has not been observed directly. The calculated IR spectra for the HCN molecule are ν2=774 cm^−1^, ν3=3428 cm^−1^ and ν1=2142 cm^−1^; the band of ν1 shows extremely low intensity (S1_fig.pdf A). As expected from prior applications of B3LYP functional to vibronic studies [[7](#_ENREF_7)], calculated and measured vibrational frequencies show almost perfect agreement.

The measured IR spectrum of benzaldehyde is available from NIST Standard Reference Database 69: NIST Chemistry WebBook

(<https://webbook.nist.gov/cgi/cbook.cgi?ID=C100527&Type=IR-SPEC&Index=1#IR-SPEC>) and exhibits the C=O stretching at 1701 cm^-1^, the aromatic C=C stretching at 1594 cm^-1^, and the phenyl aromatic C-H out-of-plane bends at 690 and 743 cm^-1^; the corresponding vibrational modes of the calculated IR spectrum for benzaldehyde are at 1707 cm^-1^, 1635, 721 cm^-1^ and 782 cm^-1^, respectively (S1_fig.pdf B).

The measured IR spectrum of nitrobenzene is available from the Spectral Database for Organic Compounds (SDBS database, <https://sdbs.db.aist.go.jp/sdbs/cgi-bin/cre_frame_disp.cgi?sdbsno=552>): the twin peaks at 1521 cm^-1^ and 1688cm^-1^, the corresponding calculated spectrum at 1526 cm^-1^ and 1648 cm^-1^ possibly representing the aromatic C=C stretching; the peak at 1347 cm^-1^ representing the N=O stretch of the nitro group and the highest peak of the whole IR spectrum of nitrobenzene, the corresponding calculated spectrum is at 1290 cm^-1^ (S1_fig.pdf C).

**Reference**

1. Choi KN, Barker EF (1932) Infrared Absorption Spectrum of Hydrogen Cyanide. Physical Review 42: 777-785.

2. Lampert H, Mikenda W, Karpfen A (1997) Molecular Geometries and Vibrational Spectra of Phenol, Benzaldehyde, and Salicylaldehyde:  Experimental versus Quantum Chemical Data. The Journal of Physical Chemistry A 101: 2254-2263.

3. Tolstorozhev GB, Skornyakov IV, Bel’kov MV, Shadyro OI, Brinkevich SD, et al. (2012) IR spectra of benzaldehyde and its derivatives in different aggregate states. Optics and Spectroscopy 113: 179-183.

4. Khaikin LS, Kochikov IV, Grikina OE, Tikhonov DS, Baskir EG (2015) IR spectra of nitrobenzene and nitrobenzene-15N in the gas phase, ab initio analysis of vibrational spectra and reliable force fields of nitrobenzene and 1,3,5-trinitrobenzene. Investigation of equilibrium geometry and internal rotation in these simplest aromatic nitro compounds with one and three rotors by means of electron diffraction, spectroscopic, and quantum chemistry data. Structural Chemistry 26: 1651-1687.

5. Stephenson CV, Coburn WC, Wilcox WS (1961) The vibrational spectra and assignments of nitrobenzene, phenyl isocyanate, phenyl isothiocyanate, thionylaniline and anisole. Spectrochimica Acta 17: 933-946.

6. Bradley MS, Bratu C (1997) Vibrational Line Profiles as a Probe of Molecular Interactions. Journal of Chemical Education 74: 553.

7. Lopez GV, Chang CH, Johnson PM, Hall GE, T.J. S (2012) What is the best DFT functional for vibronic calculations? A comparison of the calculated vibronic structure of the S1-S0 transition of phenylacetylene with cavity ringdown band intensities. J Phys Chem A 116: 6750-6758.
