# Supplementary figures and images for: Exploring the mechanism of olfactory recognition in the initial stage by modeling the emission spectrum of electron transfer
Source: PLoS One. 2020 Jan 10;15(1):e0217665. doi: 10.1371/journal.pone.0217665 (PMC6953861; doi:10.1371/journal.pone.0217665)

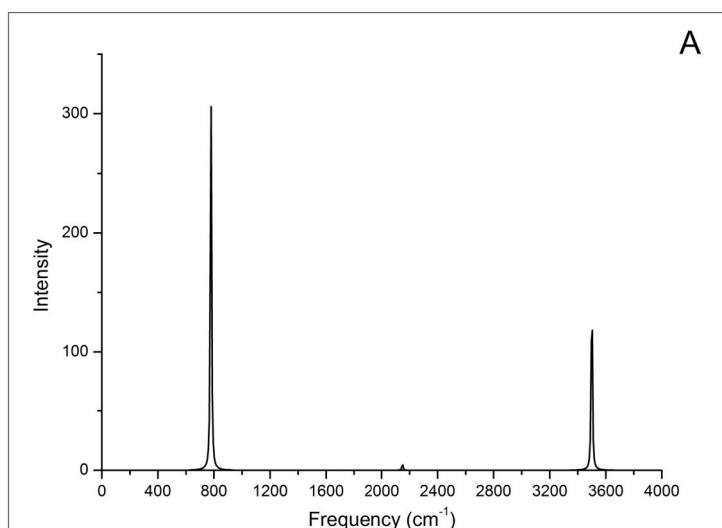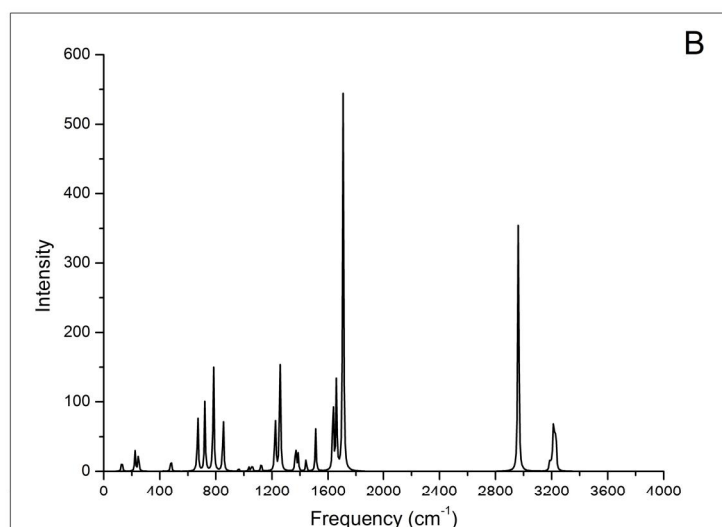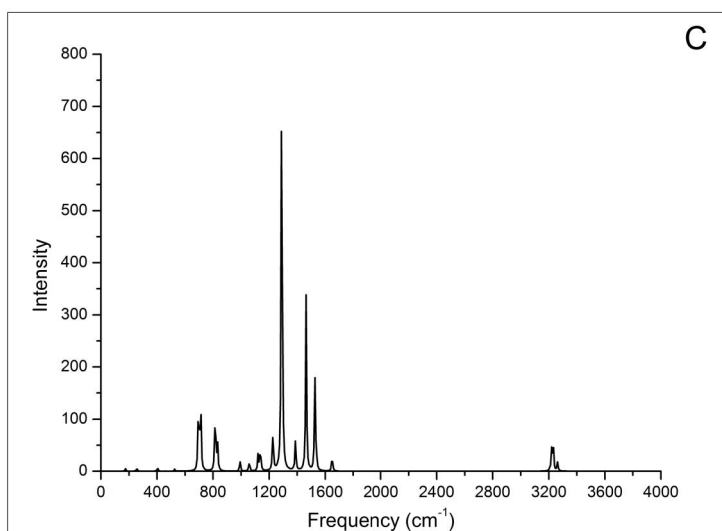

Supplement: S1 Fig — A, HCN; B, benzaldehyde; and C, nitrobenzene. (PDF) [file pone.0217665.s002.pdf]

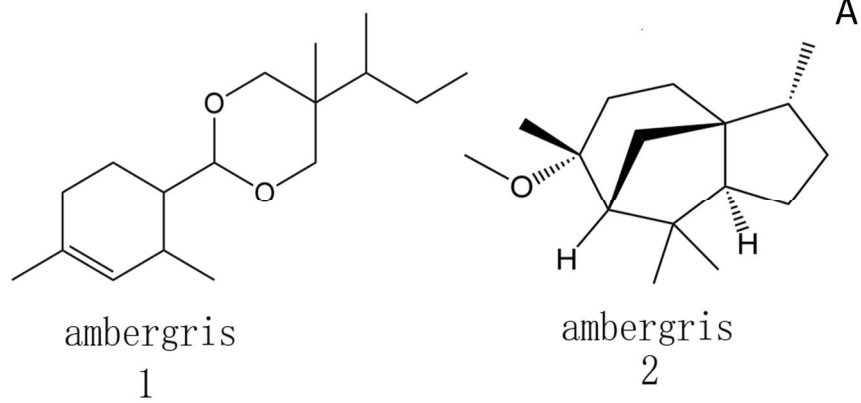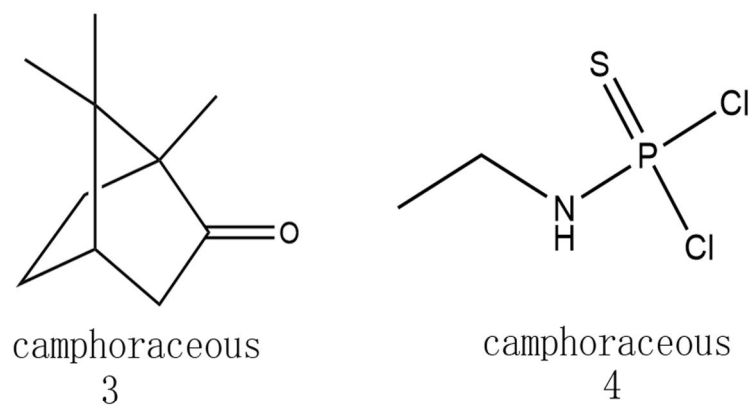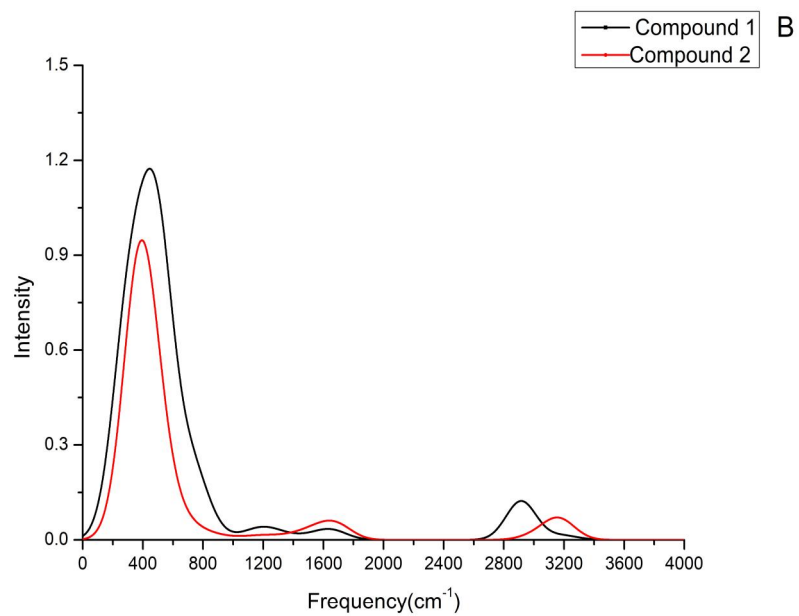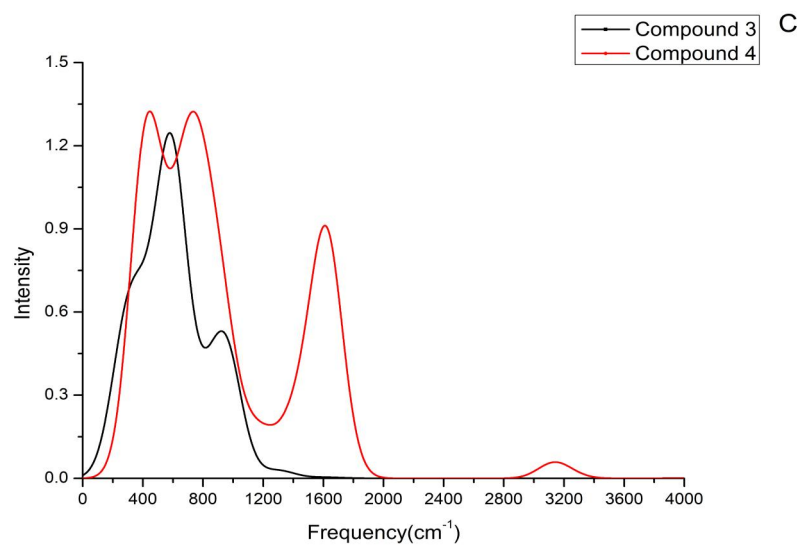

Supplement: S2 Fig — A. The structures of compound 1, karanal (2-(2,4-dimethyl-3-cyclohexen-1-yl)-5-methyl-5-(1-methylpropyl)-3-dioxane, C17H30O2); compound 2, cedramber (1H-3a,7-Methanoazulene,octahydro-6-methoxy-3,6,8,8-tetramethyl-, (3R,3aS,6R,7R,8aS)-, C16H28O); compound 3, 1,7,7-trimethylbicyclo[2.2.1]heptan-2-one (C10H16O) and compound 4, ethylphosphoramidothioic dichloride (C2H6NPSCl2). B. The ET emission spectra of karanal and cedramber. C. The ET emission spectra of 1,7,7-trimethylbicyclo[2.2.1]heptan-2-one and ethylphosphoramidothioic dichloride. (PDF) [file pone.0217665.s003.pdf]
