# Supplementary material for: Exploring the mechanism of olfactory recognition in the initial stage by modeling the emission spectrum of electron transfer
Source: PLoS One. 2020 Jan 10;15(1):e0217665. doi: 10.1371/journal.pone.0217665 (PMC6953861; doi:10.1371/journal.pone.0217665)
Supplement: S1 Table — (DOCX) [file pone.0217665.s004.docx]

**Table S1.** The Huang-Rhys Factors, and intramolecular reorganization energies, λ_i_ (eV) for each vibrational frequency, ω_i_ (cm^-1^) of benzaldehyde in its neutral and anionic states.

|  | Neutral |  |  |  | Anion |  |  |  |
| --- | --- | --- | --- | --- | --- | --- | --- | --- |
| ω_i_ |  | λ_i_ |  |  | ω_i_ |  | λ_i_ |  |
| 453 | 0.348 | 0.02 |  |  | 448 | 0.375 | 0.021 |  |
| 852 | 0.006 | 0.001 |  |  | 835 | 0.000 | 0 |  |
| 1035 | 0.079 | 0.01 |  |  | 1007 | 0.076 | 0.009 |  |
| 1222 | 0.045 | 0.007 |  |  | 1182 | 0.000 | 0 |  |
| 1226 | 0.044 | 0.007 |  |  | 1205 | 0.018 | 0.003 |  |
| 1258 | 0.079 | 0.012 |  |  | 1270 | 0.000 | 0 |  |
| 1369 | 0.003 | 0 |  |  | 1325 | 0.014 | 0.002 |  |
| 1386 | 0.000 | 0 |  |  | 1378 | 0.110 | 0.019 |  |
| 1443 | 0.042 | 0.007 |  |  | 1395 | 0.074 | 0.013 |  |
| 1512 | 0.037 | 0.007 |  |  | 1471 | 0.051 | 0.009 |  |
| 1550 | 0.030 | 0.006 |  |  | 1517 | 0.010 | 0.002 |  |
| 1636 | 0.100 | 0.02 |  |  | 1540 | 0.005 | 0.001 |  |
| 1660 | 0.253 | 0.052 |  |  | 1578 | 0.146 | 0.029 |  |
| 1707 | 0.267 | 0.057 |  |  | 1654 | 0.385 | 0.079 |  |
| 2963 | 0.004 | 0.002 |  |  | 2844 | 0.006 | 0.002 |  |
| 3232 | 0.001 | 0.001 |  |  | 3198 | 0.000 | 0 |  |
