# Supplementary material for: Exploring the mechanism of olfactory recognition in the initial stage by modeling the emission spectrum of electron transfer
Source: PLoS One. 2020 Jan 10;15(1):e0217665. doi: 10.1371/journal.pone.0217665 (PMC6953861; doi:10.1371/journal.pone.0217665)
Supplement: S2 Table — (DOCX) [file pone.0217665.s005.docx]

**Table S2.** The Huang-Rhys Factors, and intramolecular reorganization energies, λ_i_ (eV), for each vibrational frequency, ω_i_ (cm^-1^) of nitrobenzene in its neutral and anionic states.

|  | Neutral |  |  |  | Anion |  |  |  |
| --- | --- | --- | --- | --- | --- | --- | --- | --- |
| ω_i_ |  | λ_i_ |  |  | ω_i_ |  | λ_i_ |  |
| 404 | 0.010 | 0.001 |  |  | 405 | 0.014 | 0.001 |  |
| 699 | 0.248 | 0.021 |  |  | 626 | 0.400 | 0.031 |  |
| 831 | 0.208 | 0.021 |  |  | 772 | 0.111 | 0.011 |  |
| 1038 | 0.019 | 0.002 |  |  | 1009 | 0.000 | 0 |  |
| 1060 | 0.000 | 0 |  |  | 1035 | 0.006 | 0.001 |  |
| 1137 | 0.003 | 0 |  |  | 1069 | 0.243 | 0.032 |  |
| 1227 | 0.046 | 0.007 |  |  | 1200 | 0.054 | 0.008 |  |
| 1290 | 1.457 | 0.233 |  |  | 1359 | 0.778 | 0.131 |  |
| 1537 | 0.021 | 0.004 |  |  | 1524 | 0.120 | 0.023 |  |
| 1648 | 0.048 | 0.01 |  |  | 1641 | 0.162 | 0.033 |  |
| 3207 | 0.000 | 0 |  |  | 3144 | 0.001 | 0 |  |
| 3232 | 0.002 | 0.001 |  |  | 3185 | 0.001 | 0 |  |
| 3263 | 0.000 | 0 |  |  | 3250 | 0.000 | 0 |  |
