# Supplementary material for: Exploring the mechanism of olfactory recognition in the initial stage by modeling the emission spectrum of electron transfer
Source: PLoS One. 2020 Jan 10;15(1):e0217665. doi: 10.1371/journal.pone.0217665 (PMC6953861; doi:10.1371/journal.pone.0217665)
Supplement: S3 Table — (DOCX) [file pone.0217665.s006.docx]

**Table S3.** The spin population of HCN when a tunneling electron is added.

|  | spin_pop | atomic charge |
| --- | --- | --- |
| C | 0.53307 | -0.438989 |
| N | 0.50952 | -0.593551 |
| H | -0.04259 | 0.03254 |
