# Supplementary material for: Exploring the mechanism of olfactory recognition in the initial stage by modeling the emission spectrum of electron transfer
Source: PLoS One. 2020 Jan 10;15(1):e0217665. doi: 10.1371/journal.pone.0217665 (PMC6953861; doi:10.1371/journal.pone.0217665)
Supplement: S4 Table — (DOCX) [file pone.0217665.s007.docx]

**Table S4.** Duschinsky mix for selected vibrations of benzaldehyde.

|  |  |  |  | Anion |  |  |  |
| --- | --- | --- | --- | --- | --- | --- | --- |
|  | ω_i_ | 664 | 793 | 967 | 1395 | 3144 | 3175 |
|  | 721 | 0.38 |  |  |  |  |  |
|  | 783 | -0.78 | -0.5 |  |  |  |  |
|  | 888 |  | 0.47 |  |  |  |  |
|  | 963 | 0.45 | -0.67 |  |  |  |  |
| Neutral | 1016 |  |  | -0.4 |  |  |  |
|  | 1035 |  |  | -0.48 |  |  |  |
|  | 1053 |  |  | 0.73 |  |  |  |
|  | 1369 |  |  |  | -0.93 |  |  |
|  | 1386 |  |  |  | -0.62 |  |  |
|  | 1443 |  |  |  | -0.5 |  |  |
|  | 1512 |  |  |  | -0.38 |  |  |
|  | 3188 |  |  |  |  | 0.45 |  |
|  | 3199 |  |  |  |  | -0.53 | -0.34 |
|  | 3211 |  |  |  |  | -0.64 |  |
|  | 3223 |  |  |  |  | -0.33 | 0.81 |
|  | 3232 |  |  |  |  |  | 0.36 |
