# Supplementary material for: Exploring the mechanism of olfactory recognition in the initial stage by modeling the emission spectrum of electron transfer
Source: PLoS One. 2020 Jan 10;15(1):e0217665. doi: 10.1371/journal.pone.0217665 (PMC6953861; doi:10.1371/journal.pone.0217665)
Supplement: S5 Table — (DOCX) [file pone.0217665.s008.docx]

**Table S5.** Duschinsky mix for selected vibrations of nitrobenzene.

|  |  |  |  | Anion |  |  |  |  |  |  |
| --- | --- | --- | --- | --- | --- | --- | --- | --- | --- | --- |
|  | ω_i_ | 389 | 517 | 626 | 772 | 1009 | 1069 | 1359 | 3144 | 3185 |
|  | 456 | 0.89 | 0.426 |  |  |  |  |  |  |  |
|  | 694 | 0.414 | 0.848 |  |  |  |  |  |  |  |
|  | 699 |  |  | -0.9 | 0.432 |  |  |  |  |  |
|  | 831 |  |  | 0.413 | 0.879 |  |  |  |  |  |
| Neutral | 1038 |  |  |  |  | 0.879 | -0.355 |  |  |  |
|  | 1137 |  |  |  |  | 0.376 | 0.773 | 0.464 |  |  |
|  | 1290 |  |  |  |  |  | -0.437 | 0.831 |  |  |
|  | 3207 |  |  |  |  |  |  |  | 0.94 | 0.337 |
|  | 3232 |  |  |  |  |  |  |  | -0.331 | 0.938 |
