# Supplementary material for: Exploring the mechanism of olfactory recognition in the initial stage by modeling the emission spectrum of electron transfer
Source: PLoS One. 2020 Jan 10;15(1):e0217665. doi: 10.1371/journal.pone.0217665 (PMC6953861; doi:10.1371/journal.pone.0217665)
Supplement: S6 Table — (DOCX) [file pone.0217665.s009.docx]

**Table S6.** The Huang-Rhys Factors, and intramolecular reorganization energies, λ_i_ (eV) for each vibrational frequency, ω_i_ (cm^-1^) of acetophenone in its neutral and anionic states.

|  | Neutral |  |  |  | Anion |  |  |  |
| --- | --- | --- | --- | --- | --- | --- | --- | --- |
| ω_i_ |  | λ_i_ |  |  | ω_i_ |  | λ_i_ |  |
| 223 | 0.049 | 0.001 |  |  | 211 | 0.055 | 0.001 |  |
| 373 | 0.036 | 0.002 |  |  | 362 | 0.051 | 0.002 |  |
| 478 | 0.167 | 0.01 |  |  | 480 | 0.177 | 0.011 |  |
| 987 | 0.170 | 0.021 |  |  | 957 | 0.059 | 0.007 |  |
| 1036 | 0.133 | 0.017 |  |  | 1014 | 0.188 | 0.024 |  |
| 1063 | 0.007 | 0.001 |  |  | 1023 | 0.008 | 0.001 |  |
| 1120 | 0.069 | 0.01 |  |  | 1085 | 0.065 | 0.009 |  |
| 1129 | 0.028 | 0.004 |  |  | 1102 | 0.006 | 0.001 |  |
| 1223 | 0.003 | 0.001 |  |  | 1184 | 0.000 | 0 |  |
| 1236 | 0.038 | 0.006 |  |  | 1217 | 0.031 | 0.005 |  |
| 1312 | 0.138 | 0.023 |  |  | 1306 | 0.001 | 0 |  |
| 1372 | 0.017 | 0.003 |  |  | 1351 | 0.032 | 0.005 |  |
| 1437 | 0.007 | 0.001 |  |  | 1405 | 0.014 | 0.002 |  |
| 1506 | 0.024 | 0.005 |  |  | 1470 | 0.069 | 0.013 |  |
| 1513 | 0.009 | 0.002 |  |  | 1491 | 0.091 | 0.017 |  |
| 1551 | 0.044 | 0.008 |  |  | 1525 | 0.050 | 0.009 |  |
| 1633 | 0.061 | 0.012 |  |  | 1540 | 0.002 | 0 |  |
| 1659 | 0.289 | 0.059 |  |  | 1560 | 0.179 | 0.035 |  |
| 1691 | 0.185 | 0.039 |  |  | 1652 | 0.348 | 0.071 |  |
| 3051 | 0.003 | 0.001 |  |  | 2966 | 0.004 | 0.001 |  |
| 3195 | 0.001 | 0 |  |  | 3120 | 0.001 | 0.001 |  |
