# Supplementary material for: Exploring the mechanism of olfactory recognition in the initial stage by modeling the emission spectrum of electron transfer
Source: PLoS One. 2020 Jan 10;15(1):e0217665. doi: 10.1371/journal.pone.0217665 (PMC6953861; doi:10.1371/journal.pone.0217665)
Supplement: S7 Table — (DOCX) [file pone.0217665.s010.docx]

**Table S7.** The Huang-Rhys Factors, and intramolecular reorganization energies, λ_i_ (eV) for each vibrational frequency, ω_i_ (cm^-1^) of acetophenone-d8 in its neutral and anionic states.

|  | Neutral |  |  |  | Anion |  |  |  |
| --- | --- | --- | --- | --- | --- | --- | --- | --- |
| ω_i_ |  | λ_i_ |  |  | ω_i_ |  | λ_i_ |  |
| 203 | 0.037 | 0.001 |  |  | 192 | 0.041 | 0.001 |  |
| 346 | 0.034 | 0.001 |  |  | 335 | 0.051 | 0.002 |  |
| 448 | 0.145 | 0.008 |  |  | 450 | 0.159 | 0.009 |  |
| 707 | 0.006 | 0.001 |  |  | 689 | 0.000 | 0 |  |
| 837 | 0.264 | 0.027 |  |  | 811 | 0.210 | 0.021 |  |
| 854 | 0.003 | 0 |  |  | 832 | 0.006 | 0.001 |  |
| 867 | 0.030 | 0.003 |  |  | 852 | 0.006 | 0.001 |  |
| 904 | 0.020 | 0.002 |  |  | 884 | 0.063 | 0.007 |  |
| 990 | 0.092 | 0.011 |  |  | 966 | 0.079 | 0.009 |  |
| 1022 | 0.000 | 0 |  |  | 997 | 0.007 | 0.001 |  |
| 1089 | 0.000 | 0 |  |  | 1075 | 0.008 | 0.001 |  |
| 1091 | 0.012 | 0.002 |  |  | 1103 | 0.002 | 0 |  |
| 1132 | 0.019 | 0.003 |  |  | 1111 | 0.006 | 0.001 |  |
| 1262 | 0.095 | 0.015 |  |  | 1249 | 0.006 | 0.001 |  |
| 1370 | 0.022 | 0.004 |  |  | 1313 | 0.027 | 0.004 |  |
| 1377 | 0.003 | 0.001 |  |  | 1387 | 0.004 | 0.001 |  |
| 1423 | 0.114 | 0.02 |  |  | 1425 | 0.012 | 0.002 |  |
| 1596 | 0.046 | 0.009 |  |  | 1463 | 0.037 | 0.007 |  |
| 1622 | 0.293 | 0.059 |  |  | 1521 | 0.290 | 0.055 |  |
| 1680 | 0.268 | 0.056 |  |  | 1618 | 0.450 | 0.09 |  |
| 2193 | 0.005 | 0.001 |  |  | 2132 | 0.006 | 0.001 |  |
| 2350 | 0.000 | 0 |  |  | 2297 | 0.003 | 0.001 |  |
| 2356 | 0.003 | 0.001 |  |  | 2302 | 0.000 | 0 |  |
