# Supplementary material for: Exploring the mechanism of olfactory recognition in the initial stage by modeling the emission spectrum of electron transfer
Source: PLoS One. 2020 Jan 10;15(1):e0217665. doi: 10.1371/journal.pone.0217665 (PMC6953861; doi:10.1371/journal.pone.0217665)
Supplement: S8 Table — (DOCX) [file pone.0217665.s011.docx]

**Table S8.** The Huang-Rhys Factors, and intramolecular reorganization energies, λ_i_ (eV) for each vibrational frequency, ω_i_ (cm^-1^) of cyclopentadecanone in its neutral and anionic states.

|  | Neutral |  |  |  | Anionic |  |  |  |
| --- | --- | --- | --- | --- | --- | --- | --- | --- |
| ω_i_ |  | λ_i_ |  |  | ω_i_ |  | λ_i_ |  |
| 35 | 5.267 | 0.023 |  |  | 37 | 4.627 | 0.021 |  |
| 65 | 0.019 | 0 |  |  | 53 | 3.170 | 0.021 |  |
| 74 | 1.945 | 0.018 |  |  | 64 | 0.159 | 0.001 |  |
| 84 | 0.623 | 0.007 |  |  | 85 | 0.050 | 0.001 |  |
| 91 | 0.362 | 0.004 |  |  | 89 | 2.831 | 0.031 |  |
| 108 | 0.788 | 0.011 |  |  | 92 | 0.663 | 0.008 |  |
| 134 | 0.285 | 0.005 |  |  | 118 | 0.011 | 0 |  |
| 152 | 0.111 | 0.002 |  |  | 145 | 0.001 | 0 |  |
| 169 | 0.027 | 0.001 |  |  | 168 | 0.025 | 0.001 |  |
| 186 | 0.068 | 0.002 |  |  | 188 | 0.015 | 0 |  |
| 222 | 0.262 | 0.007 |  |  | 199 | 0.002 | 0 |  |
| 235 | 0.098 | 0.003 |  |  | 236 | 0.001 | 0 |  |
| 265 | 0.013 | 0 |  |  | 262 | 0.023 | 0.001 |  |
| 282 | 0.009 | 0 |  |  | 286 | 0.057 | 0.002 |  |
| 295 | 0.015 | 0.001 |  |  | 294 | 0.131 | 0.005 |  |
| 301 | 0.014 | 0.001 |  |  | 304 | 0.002 | 0 |  |
| 359 | 0.025 | 0.001 |  |  | 359 | 0.018 | 0.001 |  |
| 373 | 0.381 | 0.018 |  |  | 372 | 0.411 | 0.019 |  |
| 405 | 0.125 | 0.006 |  |  | 388 | 0.013 | 0.001 |  |
| 416 | 0.068 | 0.004 |  |  | 405 | 0.019 | 0.001 |  |
| 467 | 0.081 | 0.005 |  |  | 418 | 0.415 | 0.021 |  |
| 479 | 0.026 | 0.002 |  |  | 459 | 0.000 | 0 |  |
| 503 | 0.057 | 0.004 |  |  | 482 | 0.104 | 0.006 |  |
| 529 | 0.800 | 0.052 |  |  | 499 | 0.043 | 0.003 |  |
| 673 | 0.689 | 0.057 |  |  | 595 | 0.360 | 0.027 |  |
| 744 | 0.019 | 0.002 |  |  | 728 | 0.027 | 0.002 |  |
| 745 | 0.118 | 0.011 |  |  | 740 | 0.000 | 0 |  |
| 769 | 0.052 | 0.005 |  |  | 761 | 0.064 | 0.006 |  |
| 790 | 0.068 | 0.007 |  |  | 781 | 0.030 | 0.003 |  |
| 802 | 0.005 | 0 |  |  | 794 | 0.005 | 0.001 |  |
| 824 | 0.084 | 0.009 |  |  | 801 | 0.003 | 0 |  |
| 825 | 0.021 | 0.002 |  |  | 815 | 0.001 | 0 |  |
| 858 | 0.001 | 0 |  |  | 849 | 0.006 | 0.001 |  |
| 876 | 0.076 | 0.008 |  |  | 860 | 0.010 | 0.001 |  |
| 897 | 0.001 | 0 |  |  | 898 | 0.023 | 0.003 |  |
| 926 | 0.000 | 0 |  |  | 917 | 0.050 | 0.006 |  |
| 971 | 0.012 | 0.001 |  |  | 939 | 0.004 | 0 |  |
| 975 | 0.028 | 0.003 |  |  | 967 | 0.004 | 0 |  |
| 986 | 0.063 | 0.008 |  |  | 971 | 0.050 | 0.006 |  |
| 1004 | 0.011 | 0.001 |  |  | 994 | 0.011 | 0.001 |  |
| 1029 | 0.006 | 0.001 |  |  | 1019 | 0.037 | 0.005 |  |
| 1039 | 0.034 | 0.004 |  |  | 1025 | 0.013 | 0.002 |  |
| 1055 | 0.000 | 0 |  |  | 1039 | 0.032 | 0.004 |  |
| 1074 | 0.015 | 0.002 |  |  | 1061 | 0.003 | 0 |  |
| 1088 | 0.043 | 0.006 |  |  | 1074 | 0.008 | 0.001 |  |
| 1099 | 0.003 | 0 |  |  | 1085 | 0.027 | 0.004 |  |
| 1107 | 0.005 | 0.001 |  |  | 1095 | 0.039 | 0.005 |  |
| 1110 | 0.012 | 0.002 |  |  | 1099 | 0.003 | 0 |  |
| 1125 | 0.038 | 0.005 |  |  | 1105 | 0.051 | 0.007 |  |
| 1133 | 0.001 | 0 |  |  | 1129 | 0.005 | 0.001 |  |
| 1146 | 0.037 | 0.005 |  |  | 1141 | 0.028 | 0.004 |  |
| 1173 | 0.088 | 0.013 |  |  | 1160 | 0.012 | 0.002 |  |
| 1195 | 0.047 | 0.007 |  |  | 1171 | 0.088 | 0.013 |  |
| 1224 | 0.032 | 0.005 |  |  | 1199 | 0.028 | 0.004 |  |
| 1247 | 0.023 | 0.004 |  |  | 1239 | 0.095 | 0.015 |  |
| 1260 | 0.001 | 0 |  |  | 1245 | 0.042 | 0.007 |  |
| 1273 | 0.045 | 0.007 |  |  | 1261 | 0.042 | 0.007 |  |
| 1291 | 0.013 | 0.002 |  |  | 1284 | 0.030 | 0.005 |  |
| 1307 | 0.020 | 0.003 |  |  | 1294 | 0.036 | 0.006 |  |
| 1315 | 0.004 | 0.001 |  |  | 1307 | 0.000 | 0 |  |
| 1330 | 0.007 | 0.001 |  |  | 1318 | 0.005 | 0.001 |  |
| 1340 | 0.003 | 0.001 |  |  | 1327 | 0.084 | 0.014 |  |
| 1347 | 0.013 | 0.002 |  |  | 1329 | 0.180 | 0.03 |  |
| 1349 | 0.000 | 0 |  |  | 1340 | 0.016 | 0.003 |  |
| 1355 | 0.015 | 0.002 |  |  | 1343 | 0.009 | 0.001 |  |
| 1356 | 0.004 | 0.001 |  |  | 1349 | 0.028 | 0.005 |  |
| 1359 | 0.001 | 0 |  |  | 1355 | 0.007 | 0.001 |  |
| 1393 | 0.016 | 0.003 |  |  | 1362 | 0.001 | 0 |  |
| 1396 | 0.000 | 0 |  |  | 1363 | 0.009 | 0.002 |  |
| 1404 | 0.005 | 0.001 |  |  | 1389 | 0.018 | 0.003 |  |
| 1408 | 0.022 | 0.004 |  |  | 1392 | 0.011 | 0.002 |  |
| 1411 | 0.055 | 0.01 |  |  | 1395 | 0.012 | 0.002 |  |
| 1415 | 0.010 | 0.002 |  |  | 1399 | 0.000 | 0 |  |
| 1419 | 0.001 | 0 |  |  | 1410 | 0.008 | 0.001 |  |
| 1425 | 0.008 | 0.001 |  |  | 1415 | 0.000 | 0 |  |
| 1430 | 0.007 | 0.001 |  |  | 1416 | 0.000 | 0 |  |
| 1513 | 0.010 | 0.002 |  |  | 1434 | 0.482 | 0.086 |  |
| 1516 | 0.042 | 0.008 |  |  | 1493 | 0.001 | 0 |  |
| 1532 | 0.003 | 0.001 |  |  | 1521 | 0.000 | 0 |  |
| 1537 | 0.000 | 0 |  |  | 1529 | 0.007 | 0.001 |  |
| 1538 | 0.000 | 0 |  |  | 1531 | 0.003 | 0.001 |  |
| 1540 | 0.004 | 0.001 |  |  | 1533 | 0.021 | 0.004 |  |
| 1541 | 0.009 | 0.002 |  |  | 1535 | 0.001 | 0 |  |
| 1710 | 0.977 | 0.207 |  |  | 1559 | 0.001 | 0 |  |
| 3008 | 0.000 | 0 |  |  | 2847 | 0.020 | 0.007 |  |
| 3012 | 0.000 | 0 |  |  | 2952 | 0.002 | 0.001 |  |
| 3012 | 0.000 | 0 |  |  | 2955 | 0.004 | 0.001 |  |
| 3018 | 0.001 | 0.001 |  |  | 2985 | 0.001 | 0 |  |
| 3019 | 0.003 | 0.001 |  |  | 2986 | 0.000 | 0 |  |
| 3031 | 0.002 | 0.001 |  |  | 3004 | 0.001 | 0 |  |
| 3038 | 0.005 | 0.002 |  |  | 3006 | 0.000 | 0 |  |
| 3042 | 0.001 | 0 |  |  | 3007 | 0.001 | 0.001 |  |
| 3045 | 0.004 | 0.002 |  |  | 3010 | 0.000 | 0 |  |
| 3051 | 0.001 | 0 |  |  | 3021 | 0.004 | 0.001 |  |
| 3070 | 0.001 | 0.001 |  |  | 3047 | 0.000 | 0 |  |
| 3113 | 0.002 | 0.001 |  |  | 3105 | 0.000 | 0 |  |
| 3136 | 0.002 | 0.001 |  |  | 3110 | 0.000 | 0 |  |
