# Supplementary material for: Exploring the mechanism of olfactory recognition in the initial stage by modeling the emission spectrum of electron transfer
Source: PLoS One. 2020 Jan 10;15(1):e0217665. doi: 10.1371/journal.pone.0217665 (PMC6953861; doi:10.1371/journal.pone.0217665)
Supplement: S9 Table — (DOCX) [file pone.0217665.s012.docx]

**Table S9**. The Huang-Rhys Factors, and intramolecular reorganization energies, λ_i_ (eV) for each vibrational frequency, ω_i_ (cm^-1^) of cyclopentadecanone-d28 in its neutral and anionic states.

|  | Neutral |  |  |  | Anion |  |  |  |
| --- | --- | --- | --- | --- | --- | --- | --- | --- |
| ω_i_ |  | λ_i_ |  |  | ω_i_ |  | λ_i_ |  |
| 34 | 5.300 | 0.022 |  |  | 35 | 1.471 | 0.006 |  |
| 56 | 0.001 | 0 |  |  | 40 | 6.347 | 0.031 |  |
| 63 | 3.279 | 0.026 |  |  | 62 | 0.320 | 0.002 |  |
| 73 | 0.228 | 0.002 |  |  | 78 | 0.227 | 0.002 |  |
| 79 | 0.006 | 0 |  |  | 82 | 2.870 | 0.029 |  |
| 96 | 0.717 | 0.009 |  |  | 88 | 0.654 | 0.007 |  |
| 111 | 0.111 | 0.002 |  |  | 104 | 0.203 | 0.003 |  |
| 124 | 0.145 | 0.002 |  |  | 114 | 0.004 | 0 |  |
| 128 | 0.254 | 0.004 |  |  | 124 | 0.000 | 0 |  |
| 150 | 0.100 | 0.002 |  |  | 146 | 0.003 | 0 |  |
| 163 | 0.025 | 0.001 |  |  | 163 | 0.017 | 0 |  |
| 196 | 0.228 | 0.006 |  |  | 182 | 0.003 | 0 |  |
| 201 | 0.078 | 0.002 |  |  | 201 | 0.006 | 0 |  |
| 224 | 0.029 | 0.001 |  |  | 230 | 0.001 | 0 |  |
| 240 | 0.001 | 0 |  |  | 236 | 0.135 | 0.004 |  |
| 253 | 0.105 | 0.003 |  |  | 254 | 0.000 | 0 |  |
| 306 | 0.014 | 0.001 |  |  | 304 | 0.037 | 0.001 |  |
| 330 | 0.260 | 0.011 |  |  | 330 | 0.123 | 0.005 |  |
| 349 | 0.155 | 0.007 |  |  | 336 | 0.125 | 0.005 |  |
| 361 | 0.132 | 0.006 |  |  | 348 | 0.022 | 0.001 |  |
| 382 | 0.098 | 0.005 |  |  | 367 | 0.335 | 0.015 |  |
| 394 | 0.200 | 0.01 |  |  | 385 | 0.147 | 0.007 |  |
| 415 | 0.039 | 0.002 |  |  | 395 | 0.178 | 0.009 |  |
| 477 | 0.647 | 0.038 |  |  | 415 | 0.005 | 0 |  |
| 548 | 0.033 | 0.002 |  |  | 540 | 0.346 | 0.023 |  |
| 578 | 0.214 | 0.015 |  |  | 550 | 0.161 | 0.011 |  |
| 582 | 0.069 | 0.005 |  |  | 574 | 0.004 | 0 |  |
| 603 | 0.246 | 0.018 |  |  | 583 | 0.003 | 0 |  |
| 633 | 0.005 | 0 |  |  | 629 | 0.019 | 0.001 |  |
| 657 | 0.052 | 0.004 |  |  | 645 | 0.020 | 0.002 |  |
| 667 | 0.015 | 0.001 |  |  | 658 | 0.001 | 0 |  |
| 672 | 0.101 | 0.008 |  |  | 664 | 0.011 | 0.001 |  |
| 708 | 0.001 | 0 |  |  | 694 | 0.007 | 0.001 |  |
| 713 | 0.248 | 0.022 |  |  | 705 | 0.022 | 0.002 |  |
| 729 | 0.020 | 0.002 |  |  | 726 | 0.020 | 0.002 |  |
| 753 | 0.084 | 0.008 |  |  | 749 | 0.002 | 0 |  |
| 776 | 0.000 | 0 |  |  | 767 | 0.060 | 0.006 |  |
| 789 | 0.031 | 0.003 |  |  | 781 | 0.016 | 0.002 |  |
| 798 | 0.002 | 0 |  |  | 792 | 0.256 | 0.025 |  |
| 805 | 0.010 | 0.001 |  |  | 799 | 0.012 | 0.001 |  |
| 814 | 0.017 | 0.002 |  |  | 803 | 0.025 | 0.003 |  |
| 823 | 0.019 | 0.002 |  |  | 818 | 0.012 | 0.001 |  |
| 833 | 0.013 | 0.001 |  |  | 831 | 0.080 | 0.008 |  |
| 842 | 0.016 | 0.002 |  |  | 841 | 0.031 | 0.003 |  |
| 855 | 0.008 | 0.001 |  |  | 842 | 0.000 | 0 |  |
| 869 | 0.000 | 0 |  |  | 855 | 0.023 | 0.002 |  |
| 878 | 0.067 | 0.007 |  |  | 864 | 0.006 | 0.001 |  |
| 888 | 0.006 | 0.001 |  |  | 885 | 0.032 | 0.003 |  |
| 900 | 0.015 | 0.002 |  |  | 886 | 0.067 | 0.007 |  |
| 905 | 0.001 | 0 |  |  | 894 | 0.036 | 0.004 |  |
| 931 | 0.009 | 0.001 |  |  | 914 | 0.009 | 0.001 |  |
| 950 | 0.086 | 0.01 |  |  | 923 | 0.011 | 0.001 |  |
| 966 | 0.022 | 0.003 |  |  | 929 | 0.055 | 0.006 |  |
| 983 | 0.078 | 0.009 |  |  | 974 | 0.015 | 0.002 |  |
| 996 | 0.034 | 0.004 |  |  | 985 | 0.018 | 0.002 |  |
| 1003 | 0.126 | 0.016 |  |  | 996 | 0.032 | 0.004 |  |
| 1005 | 0.001 | 0 |  |  | 999 | 0.002 | 0 |  |
| 1015 | 0.064 | 0.008 |  |  | 1006 | 0.005 | 0.001 |  |
| 1016 | 0.004 | 0.001 |  |  | 1013 | 0.000 | 0 |  |
| 1029 | 0.015 | 0.002 |  |  | 1025 | 0.002 | 0 |  |
| 1044 | 0.001 | 0 |  |  | 1031 | 0.010 | 0.001 |  |
| 1051 | 0.007 | 0.001 |  |  | 1042 | 0.001 | 0 |  |
| 1062 | 0.159 | 0.021 |  |  | 1057 | 0.000 | 0 |  |
| 1067 | 0.047 | 0.006 |  |  | 1063 | 0.022 | 0.003 |  |
| 1081 | 0.024 | 0.003 |  |  | 1070 | 0.189 | 0.025 |  |
| 1122 | 0.018 | 0.002 |  |  | 1110 | 0.003 | 0 |  |
| 1130 | 0.052 | 0.007 |  |  | 1120 | 0.004 | 0.001 |  |
| 1133 | 0.021 | 0.003 |  |  | 1123 | 0.021 | 0.003 |  |
| 1136 | 0.007 | 0.001 |  |  | 1127 | 0.002 | 0 |  |
| 1142 | 0.001 | 0 |  |  | 1134 | 0.059 | 0.008 |  |
| 1147 | 0.006 | 0.001 |  |  | 1140 | 0.012 | 0.002 |  |
| 1151 | 0.004 | 0.001 |  |  | 1145 | 0.000 | 0 |  |
| 1156 | 0.070 | 0.01 |  |  | 1151 | 0.001 | 0 |  |
| 1166 | 0.004 | 0.001 |  |  | 1161 | 0.021 | 0.003 |  |
| 1169 | 0.019 | 0.003 |  |  | 1163 | 0.003 | 0 |  |
| 1187 | 0.000 | 0 |  |  | 1176 | 0.008 | 0.001 |  |
| 1201 | 0.001 | 0 |  |  | 1192 | 0.014 | 0.002 |  |
| 1210 | 0.011 | 0.002 |  |  | 1196 | 0.000 | 0 |  |
| 1262 | 0.020 | 0.003 |  |  | 1246 | 0.001 | 0 |  |
| 1699 | 1.039 | 0.219 |  |  | 1397 | 0.797 | 0.138 |  |
| 2188 | 0.000 | 0 |  |  | 2078 | 0.028 | 0.007 |  |
| 2191 | 0.000 | 0 |  |  | 2146 | 0.010 | 0.003 |  |
| 2192 | 0.000 | 0 |  |  | 2155 | 0.003 | 0.001 |  |
| 2201 | 0.002 | 0.001 |  |  | 2176 | 0.002 | 0.001 |  |
| 2208 | 0.005 | 0.001 |  |  | 2192 | 0.000 | 0 |  |
| 2215 | 0.016 | 0.004 |  |  | 2195 | 0.001 | 0 |  |
| 2216 | 0.009 | 0.002 |  |  | 2217 | 0.000 | 0 |  |
| 2305 | 0.002 | 0.001 |  |  | 2297 | 0.001 | 0 |  |
